# Enhancing MyD88 oligomerization is one important mechanism by which IBDV VP2 induces inflammatory response

**DOI:** 10.1371/journal.ppat.1012985

**Published:** 2025-03-11

**Authors:** Mengmeng Huang, Mengmeng Xu, Jingzhe Han, Erjing Ke, Xinxin Niu, Yulong Zhang, Guodong Wang, Hangbo Yu, Runhang Liu, Suyan Wang, Yongzhen Liu, Yuntong Chen, Jinze Han, Ziwen Wu, Hongyu Cui, Yanping Zhang, Yulu Duan, Yulong Gao, Xiaole Qi

**Affiliations:** 1 Avian Immunosuppressive Diseases Division, State Key Laboratory for Animal Disease Control and Prevention, Harbin Veterinary Research Institute, the Chinese Academy of Agricultural Sciences, Harbin, China; 2 World Organization for Animal Health (WOAH) Reference Laboratory for Infectious Bursal Disease, Harbin Veterinary Research Institute, the Chinese Academy of Agricultural Sciences, Harbin, China; 3 Tianjin Key Laboratory of Agricultural Animal Breeding and Healthy Husbandry, College of Animal Science and Veterinary Medicine, Tianjin Agricultural University, Tianjin, China; 4 Jiangsu Co-Innovation Center for the Prevention and Control of Important Animal Infectious Disease and Zoonosis, Yangzhou University, Yangzhou, China; Pirbright Institute, UNITED KINGDOM OF GREAT BRITAIN AND NORTHERN IRELAND

## Abstract

The inflammatory response is an essential component of innate immunity to defense against pathogens. Infectious bursal disease (IBD) is the most important immunosuppressive disease in chickens and is caused by the infectious bursal disease virus (IBDV). Acute inflammation is a typical pathogenic process for IBD, however, the underlying mechanism is not clear. Here, we report that IBDV induces obvious inflammatory response *in vivo* and *in vitro*. Furthermore, viral VP2 is identified as an important inflammatory stimulus. It is observed that IBDV VP2 can activate NF-κB signaling pathway and then increase IL-1β production. In detail, IBDV VP2 interacts with myeloid differentiation primary response gene 88 (MyD88), potentiates the oligomerization of MyD88 and assembly of MyD88 complex, which is one important element leading to NF-κB signaling pathway activation and IL-1β production increase. More meaningfully, residues 253/284 of viral VP2 are significantly involved in IBDV-induced inflammatory response through modulating the interaction strength between VP2 and MyD88 and the following MyD88-NF-κB-IL-1β signaling pathway. This study reveals one molecular mechanism that trigger inflammation during IBDV infection, which is of great significance for a deeper understanding of the pathogenic mechanisms of IBDV.

## Author summary

The inflammatory response is an important pathogenic process for infectious bursal disease (IBD). Whereas, the in-depth mechanisms by which the infectious bursal disease virus (IBDV) induces inflammation are still poorly understood. In this study, we show that IBDV infection promotes IL-1β production and activates NF-κB signaling pathway and identify that IBDV VP2 targets to the myeloid differentiation primary response gene 88 (MyD88), promotes MyD88 complex assembly and MyD88 oligomerization, which highlight the role of IBDV VP2 in modulating MyD88-NF-κB signaling network in inflammation during IBDV infection. This study contributes to a better understanding of the pathogenesis of IBDV infection.

## Introduction

Innate immunity is critical host defense system of resisting pathogens invasion, which efficiently recognizes the pathogen-associated molecular patterns (PAMPs) of pathogens to activate cellular pattern recognition receptors (PRRs) to induce innate immune response [1,2]. A wide array of microbial components, such as proteins, could lead to the activation of the PRRs such as toll-like receptors (TLRs) to trigger innate immune defense system [3,4]. Upon ligand binding, TLRs bind to MyD88 through the common toll/interleukin-1 receptor (TIR) domain, which can change conformation and enhance the proximity of TIR domains. The death domain (DD) domain of MyD88 couples enzymatic activity with self-stacking and produces plenty of helical oligomers. Six MyD88 DDs provide ring-like platforms for four IRAK4 DDs and four IRAK2 DDs combination in a helical assembly to initiate MyD88 oligomerization and activate myddosome signaling [5–8]. Another protein containing the TIR domain is the TIR-domain-containing adaptor protein (TIRAP), which possesses a lipid-binding motif that facilitates the association of MyD88 with TLRs to form myddosome protein complex. Several kinases, including the E3 ubiquitin ligase tumor necrosis factor receptor associated factor 6 (TRAF6) are recruited following myddosome activation. Subsequently, IκBα disinhibits NF-κB leading to the translocation of p65 and p50 subunits of NF-κB to nucleus and initiates the production of pro-inflammatory cytokines [9–11].

The inflammatory response, as a defense mechanism against viral infection, is a critical host defense machinery that limits viral propagation for virus clearance during innate immunity [12–14]. IBD is the most important immunosuppressive disease in chickens caused by IBDV [15,16]. IBDV is the typical member of the *Birnaviridae* family with a genome of double strand RNA (dsRNA) [17–19]. The main target cells of the virus are immature B lymphocytes, macrophages, and monocytes [20,21]. The IBDV genome comprises segment A and segment B. Segment A encodes viral proteins VP2, VP3, VP4, and VP5. Segment B encodes the RNA-dependent RNA polymerase VP1 [22–24]. IBDV is a non-enveloped virus, of which VP2 is the only capsid protein that contains the major antigenic sites which responsible for neutralizing antibodies induction and involves in the virulence, cell tropism, and pathogenic phenotype [25–28], and the acute inflammation is the typical pathogenic process [29–32]. Inoculating chicken with very virulent IBDV (vvIBDV) causes severe disease and is associated with a significant up-regulation of pathways involved in inflammation [31]. Additionally, necrosis and B cells decreased, and inflammatory cytokine level increased in the bursa tissue infected with vvIBDV, resulting in immune injury in chickens [32]. Although previous studies have demonstrate that IBDV infection increases the transcription levels of NF-κB, TNF-α, and IL-1β [33–37], the deep mechanism by which IBDV infection induces inflammation is unknown.

In the current study, we clarified whether and how IBDV VP2 promotes NF-κB signaling pathway activation leading to IL-1β secretion. Specifically, we discovered that the VP2 of IBDV can increase MyD88 oligomerization and trigger MyD88 complex assembly which is required for the activation of the pro-inflammatory response. This work elucidates one key mechanism of IBDV-induced inflammation.

## Results

### IBDV infection induces severe inflammation

**Fig 1:**
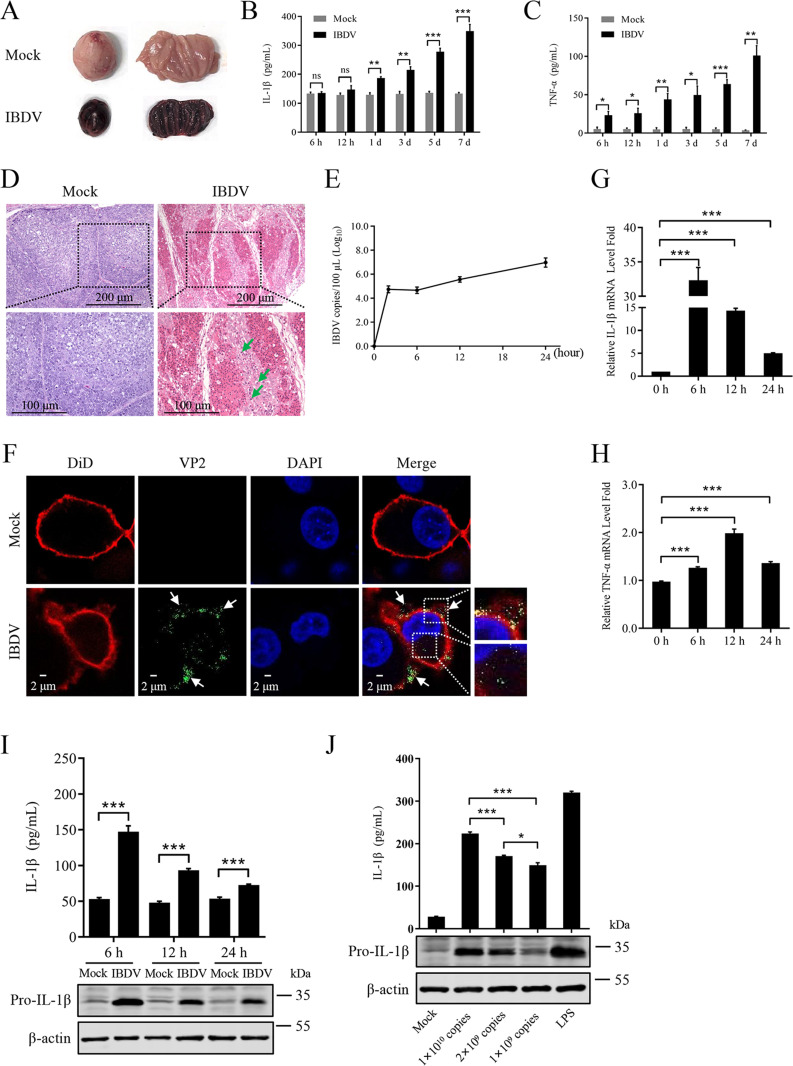
IBDV infection induces inflammatory response *in vivo* and *in vitro.* (A) Macroscopic lesions of the bursa of vvIBDV-infected chicken at 3 dpi. (B, C) Protein abundance of IL-1β (B) and TNF-α (C) in serums at indicated times post-infection were detected by ELISA. (D) Representative H&E stained analysis of the bursa sections of mock and vvIBDV infected chickens at 3 dpi. The lymphoid follicles which infected with vvIBDV were severely atrophied with hemorrhage, lymphocytes depletion, and inflammatory cells infiltration including macrophages (green arrow). (E) The vvIBDV growth dynamic in HD11 cells was analysed by RT-qPCR. (F) The vvIBDV subcellular localization. HD11 cells were incubated with vvIBDV (1×10^10^ copies/1×10^6^ cells) for 2 h. The vvIBDVs were stained as green color and the engulfed vvIBDVs by cell pseudopodia were highlighted with the white arrows and white boxes. (G, H) Effect of vvIBDV infection on IL-1β or TNF-α gene expression in HD11 cells. HD11 cells were infected with vvIBDV (1×10^10^ copies/1×10^6^ cells), and the mRNA levels of IL-1β (G) or TNF-α (H) were assessed by RT-qPCR at 0, 6, 12, 24 hpi. (I, J) Effect of vvIBDV infection on IL-1β production in HD11 cells. In the HD11 infection experiments, the IL-1β in the cell supernatants at indicated times post infection were detected by ELISA, and the pro-IL-1β in the cell protein extracts were measured by immunoblot analysis (I). In addition, different infection doses of vvIBDV on IL-1β production in HD11 cells at 6 h were detected by ELISA and immunoblot analysis (J). HD11 cells stimulated with LPS (1 μg/mL) was measured at 6 h after stimulating as a control. All data are representative of at least three independent experiments. Graphs show mean ± SD, n = 3, * P < 0.05, *** P < 0.001.

Animal experiment showed that the infection of vvIBDV HLJ0504 strain caused severe acute damage to the bursa of chickens. At 3 day post-infection (dpi), all detected bursa showed visibly atrophied with inflammatory mucus secretion, some of them even presented a typical “purple grape” like appearance due to severe bleeding (Fig 1A). Furthermore, the microscopic pathological analysis showed that the bursa lymphoid follicles of infected chicken were obviously atrophied with severely lymphocytes depletion; and a large number of inflammatory cells infiltration including macrophages were also observed (Fig 1D). In addition, in the serum of infected chickens, higher levels of IL-1β (Fig 1B) compared to mock group were detected from 1 dpi to 7 dpi, and higher levels of TNF-α (Fig 1C) compared to mock group were detected from 6 hour post-infection (hpi) to 7 dpi. These results indicate that IBDV infection could induce severe inflammation response *in vivo*.

Macrophages play important roles in initiating and regulating inflammatory responses [38]. In order to investigate the molecular mechanism by which IBDV triggers an inflammatory response, the infection experiment was performed using chicken macrophage cells HD11 by IBDV. HD11 cells were incubated with the vvIBDV HLJ0504 strain (1×10^10^ copies/1×10^6^ cells), and the number of IBDV copies gradually increased in a time-dependent manner at 2 hpi-24 hpi (Fig 1E). The confocal microscopy experiment results showed that, at 2 h post-incubation, IBDV particles were engulfed by macrophages and entered the interior of HD11 cells (Fig 1F). Next, whether IBDV promotes the up-regulation of pro-inflammatory cytokines IL-1β and TNF-α was elucidated. HD11 cells were infected with vvIBDV HLJ0504 strain or stimulated by lipopolysaccharide (LPS) for 0-12 h and the mRNA levels of IL-1β and TNF-α were assessed with RT-qPCR experiments. The results of RT-qPCR revealed that IBDV induced inflammatory response in HD11 cells, as were evident from higher levels of IL-1β (Fig 1G) and TNF-α (Fig 1H) compared to the mock control. Besides, the cell lysates were collected to analyse the pro-IL-1β production by immunoblot analyses and the cell supernatants were collected to analyse IL-1β levels by ELISA. The results showed that the protein levels of IL-1β increased after IBDV infection in HD11 cells (Fig 1I), of which this phenomenon is dose-dependent (Fig 1J). These results indicate that IBDV infection induces severe inflammatory response *in vitro*.

### IBDV capsid protein VP2 can activate the inflammatory response

**Fig 2:**
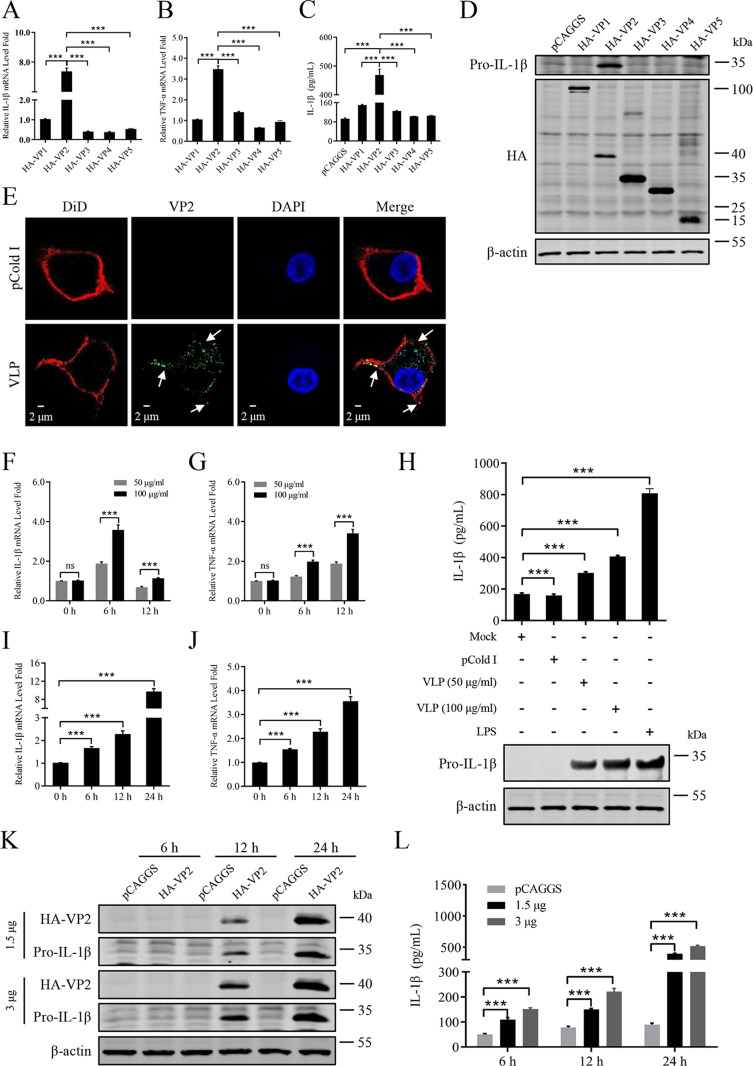
IBDV capsid protein VP2 can activate the inflammatory response. (A, B) Effect of viral VP1, VP2, VP3, VP4, and VP5 on IL-1β or TNF-α gene expression. HD11 cells were transfected with the recombinant plasmid expressing viral protein fusing HA-tag (3 μg/1×10^6^ cells) for 24 h, and levels of IL-1β (A) or TNF-α (B) mRNA were assessed by RT-qPCR. (C, D) Effect of viral VP1, VP2, VP3, VP4, and VP5 on IL-1β production. HD11 cells were transfected with the recombinant plasmid expressing viral protein fusing HA-tag (3 μg/1×10^6^ cells) for 24 h, and IL-1β in the cell supernatant were measured by ELISA (C), and pro-IL-1β in the total cell protein extracts were resolved by immunoblot analysis (D). (E) VLP of VP2 is engulfed by HD11 cells. HD11 cells were incubated with VLP of VP2 (100 μg/mL) for 2 h. VLP staining using IBDV VP2 monoclonal antibody was showed as green color and the engulfed VLP by cell pseudopodia was highlighted with the white arrows. (F, G) Effect of VLP incubation on IL-1β or TNF-α gene expression. HD11 cells were incubated with VLP (50 μg/mL or 100 μg/mL) for 0, 6, 12 h, and the IL-1β (F) or TNF-α (G) mRNA were assessed by RT-qPCR. (H) Effect of VLP incubation on IL-1β production. HD11 cells were incubated with VLP (50 μg/mL and 100 μg/mL) for 6 h or stimulated with LPS (1 μg/mL) for 6 h, the pro-IL-1β in the total cell protein extracts were detected by immunoblot analysis with specific antibodies shown on the left, and IL-1β in the cell supernatants were measured by ELISA. (I, J) Effect of viral VP2 on IL-1β or TNF-α gene expression. HD11 cells were transfected with the plasmid expressing HA-VP2 (3 μg/1×10^6^ cells) for 0, 6, 12 and 24 h, and levels of IL-1β (I) or TNF-α (J) mRNA were assessed by RT-qPCR. (K, L) Effect of VP2 on IL-1β production. HD11 cells were transfected with the plasmid expressing HA-VP2 (1.5 μg/1×10^6^ cells and 3 μg/1×10^6^ cells) for 6, 12 and 24 h, and pro-IL-1β in the total cell protein extracts were resolved by immunoblot analysis (K), and IL-1β in the cell supernatant were measured by ELISA (L). HD11 cells stimulated with LPS (1 μg/mL) was used as a control. All data are representative of at least three independent experiments. Graphs show mean ± SD, n = 3, *** P < 0.001.

The viral capsid protein is located on the outermost surface of the viral particle and has been reported to play an important role in triggering the inflammatory response in some viruses [39]. To explore whether the capsid protein VP2 of IBDV is involved in the inflammatory response, we assessed the effect of overexpression of VP1, VP2, VP3, VP4, and VP5 on activating inflammatory response in HD11 cells using RT-qPCR. The results showed that the genes expression of IL-1β (Fig 2A) and TNF-α (Fig 2B) had the strongest increase in VP2-overexpressed HD11 cells. Correspondingly, the ELISA (Fig 2C) and immunoblot (Fig 2D) results showed that the protein levels of IL-1β were increased most in VP2-overexpressed HD11 cells. Next, we tested whether the purified viral-like particle (VLP) composed of VP2 of the vvIBDV HLJ0504 strain could activate inflammatory responses. At 2 h post-incubation of HD11 cells with VLP (100 μg/ml), VLP was phagocytosed into the cytoplasm of HD11 cells (Fig 2E). Subsequently, HD11 cells were incubated with purified VLP for 0, 6, and 12 h. After treatment, the cell suspensions were collected to analyse IL-1β and TNF-α levels by RT-qPCR. RT-qPCR results showed that IL-1β (Fig 2F) and TNF-α (Fig 2G) mRNA levels in HD11 cells were effectively up-regulated by VLP, of which this phenomenon is dose-dependent. Consistently, the production of IL-1β in cell lysates detected by immunoblot analysis and in cell supernatants detected by ELISA were also be up-regulated (Fig 2H).

Subsequently, we assessed the ability of VP2 overexpression on activating inflammatory response in HD11 cells by RT-qPCR. The results showed that the genes expression of IL-1β (Fig 2I) and TNF-α (Fig 2J) was increased in VP2-overexpressed HD11 cells in a time-dependent manner. Correspondingly, the immunoblot (Fig 2K) and ELISA (Fig 2L) results showed that the protein levels of IL-1β were increased in VP2-overexpressed HD11 cells in both time-dependent and dose-dependent manner. Collectively, these results indicate that IBDV capsid protein VP2 is an important inflammatory stimulus.

### IBDV VP2 promotes the NF-κB signaling pathway activation associating with inflammatory response

**Fig 3:**
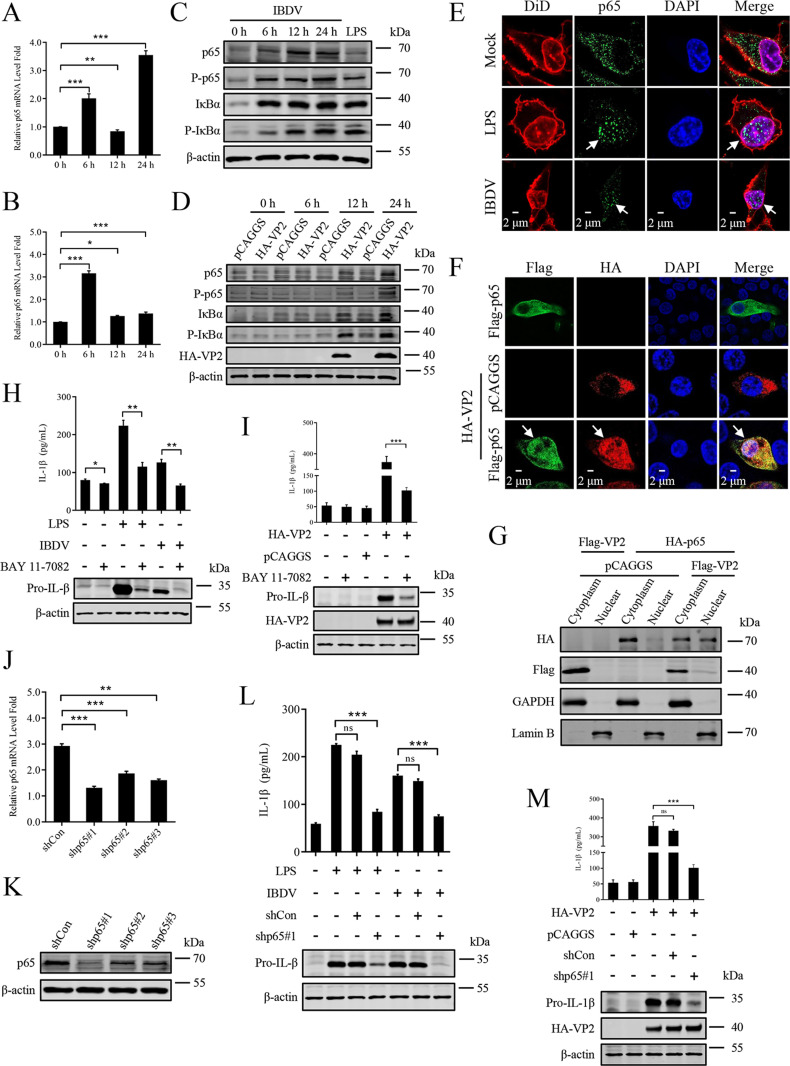
IBDV VP2 promotes the NF- **κB signaling pathway activation.** (A, B) Effect of IBDV or viral VP2 on NF-κB mediated gene expression. HD11 cells were infected with vvIBDV (1×10^10^ copies/1×10^6^ cells) (A) or transfected with the plasmid expressing HA-VP2 (3 μg/1×10^6^ cells) (B) for 0, 6, 12 and 24 h, and levels of p65 mRNA were assessed by RT-qPCR. (C, D) Effect of IBDV or viral VP2 on the phosphorylation and nuclear translocation of p65. HD11 cells were infected with vvIBDV (1×10^10^ copies/1×10^6^ cells) (C) or transfected with the plasmid expressing HA-VP2 (3 μg/1×10^6^ cells) (D) for 0, 6, 12, 24 h or stimulated with LPS (1 μg/mL) (C) for 6 h, and p65 in the total cell protein extracts were detected by immunoblot analysis. (E) Effect of IBDV on the nuclear translocation of p65. HD11 cells were infected with vvIBDV (1×10^10^ copies/1×10^6^ cells) or stimulated with LPS (1 μg/mL) for 12 h, and p65 was stained using p65 monoclonal antibody. The nuclear translocation of p65 is highlighted with the white arrows. (F, G) Effect of viral VP2 on the phosphorylation and nuclear translocation of p65. HD11 cells were transfected with the plasmid expressing Flag-p65 (2 μg/1×10^6^ cells) or HA-VP2 (2 μg/1×10^6^ cells) respectively or simultaneously for 24 h, and p65 was stained using p65 monoclonal antibody. The nuclear translocation of p65 is highlighted with the white arrows (F). The cytoplasmic and nuclear extracts were detected by immunoblot analysis (G). (H) Effect of NF-κB inhibitor on IL-1β production induced by IBDV. HD11 cells mock treated or pre-treated with BAY 11-7082 (20 μM) for 6 h were mock infected, or infected with vvIBDV (1×10^10^ copies/1×10^6^ cells) in the presence or absence of drug pre-treated for 6 h and then harvested at 6 hpi, or stimulated with LPS (1 μg/mL) in the presence or absence of drug pre-treated for 6 h and then harvested at 6 hpi. The total cell protein extracts were detected by immunoblot analysis, and IL-1β in cell supernatants were measured by ELISA. (I) Effect of NF-κB inhibitor on IL-1β production induced by viral VP2. HD11 cells mock treated or pre-treated with BAY 11-7082 (20 μM) for 6 h were mock transfected, or transfected with the plasmid expressing HA-VP2 (3 μg/1×10^6^ cells) in the presence or absence of drug pre-treated for 6 h and then harvested at 24 h. Total cell protein extracts were detected by immunoblot analysis, and the protein production of IL-1β were measured by ELISA. (J, K) RT-qPCR (J) and immunoblot (K) analysis of p65 in HD11 cells untreated or treated with indicated shRNA (1 μg/1×10^5^ cells). Twenty-four hours later, cells were infected with vvIBDV (1×10^10^ copies/1×10^6^ cells) for 6 h. (L) Effect of p65 interference on IL-1β production induced by IBDV. HD11 cells mock treated or infected with vvIBDV (1×10^10^ copies/1×10^6^ cells) in the presence or absence of shRNA-mediated specific gene silence pre-treated for 24 h and then harvested at 6 hpi, or stimulated with LPS (1 μg/mL) in the presence or absence of shRNA-mediated specific gene silence pre-treated for 24 h and then harvested at 6 h. Total cell protein extracts were detected by immunoblot analysis, and the protein production of IL-1β were measured by ELISA. (M) Effect of p65 interference on IL-1β production induced by viral VP2. HD11 cells mock treated or transfected with the plasmid expressing HA-VP2 (3 μg/1×10^6^ cells) in the presence or absence of shRNA-mediated specific gene silence pre-treated for 24 h and harvested at 24 h post-transfection. Total cell protein extracts were detected by immunoblot analysis, and the protein production of IL-1β were measured by ELISA. HD11 cells stimulated with LPS (1 μg/mL) was used as a control. All data are representative of at least three independent experiments. Graphs show mean ± SD, n = 3, * P < 0.05, ** P < 0.01, *** P < 0.001.

NF-κB is an important family of transcription factors that plays a crucial role in regulating inflammation and immune responses [40,41]. Once NF-κB signaling pathway is activated, IκBα is phosphorylated and degraded, which leads to the NF-κB subunits p65 phosphorylated and translocation from cytoplasm to nucleus and then initiates expression of a large number of pro-inflammatory genes including IL-1β [42–44]. To explore how IBDV and its VP2 regulate the NF-κB signaling pathway, we first focused on the effect on p65 transcription during IBDV infection or VP2 overexpression. HD11 cells were infected with IBDV or transfected with plasmid expressing HA-VP2 for 0-24 h and the p65 expression was monitored by RT-qPCR. IBDV infection induced significant up-regulation of p65 mRNA levels, which was approximately 3.6-fold (24 hpi) higher than that at 0 hpi (Fig 3A). In addition, viral VP2 overexpression also induced significant up-regulation of p65 mRNA compared to that at 0 h (Fig 3B).

Furthermore, the phosphorylation of IκBα and p65 during IBDV infection or VP2 overexpression was tested. The results showed that the phosphorylation levels of IκBα and p65 increased in a time-dependent manner both in IBDV-infected (Fig 3C) and VP2-overexpressed (Fig 3D) HD11 cells. The nuclear translocation of p65 is a hallmark of NF-κB signaling pathway activation. The confocal microscopy experiments results showed that p65 presented significant aggregation in the nucleus of IBDV-infected HD11 cells (Fig 3E), a phenomenon similar to that obtained in VP2-overexpressed HD11 cells (Fig 3F and 3G). These results indicate that IBDV and viral VP2 can increase the phosphorylation of IκBα and p65, leading to the activation of NF-κB signaling pathway.

Subsequently, we assessed whether BAY 11-7082 (NF-κB inhibitor) inhibited pro-inflammatory response mediated by NF-κB signaling pathway during IBDV infection. IL-1β production was monitored by immunoblot and ELISA analyses. The immunoblot and ELISA results showed that IL-1β were notably down-regulated (~50%) in IBDV-infected or LPS-stimulated HD11 cells following BAY 11-7082 incubation (Fig 3H). BAY 11-7082 incubation also inhibit IL-1β production induced by VP2 overexpression in HD11 cells (Fig 3I). In addition, the short hairpin RNA (shRNA) was designed to silence p65 expression in HD11 cells and its impact on IL-1β production was investigated. Firstly, with RT-qPCR (Fig 3J) and immunoblot (Fig 3K) experiments, three shRNA targeting p65 was screened and the interference efficiency of shp65#1 (knockdown ≥ 60%) in HD11 cells during IBDV infection was the best. With the silence of p65 using shp65#1, it was observed that the production of IL-1β significantly decreased in HD11 cells compared with that in control cells infected with IBDV or stimulated with LPS (Fig 3L). Also, we noted remarkable reduction in IL-1β production in VP2-overexpressed HD11 cells because of p65 inhibition (Fig 3I) and knockdown (Fig 3M). These results suggest that IBDV and viral VP2 significantly promote the NF-κB signaling pathway and then induce the pro-inflammatory response in HD11 cell.

### MyD88 is associated with NF-κB-dependent inflammatory response activated by IBDV

**Fig 4:**
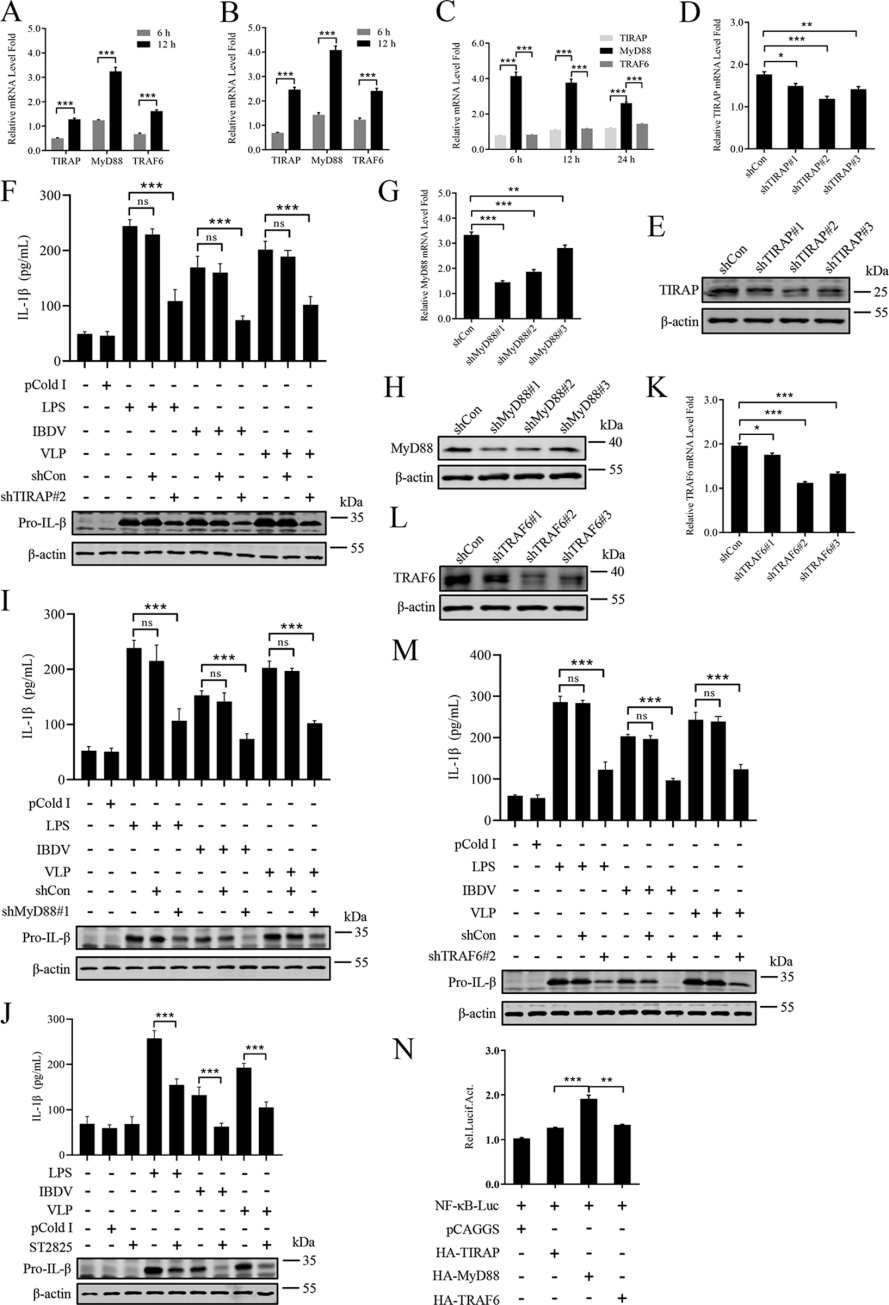
MyD88 is associated with NF- **κB-dependent inflammatory response activated by IBDV.** (A-C) Effect of IBDV or VLP or viral VP2 on TIRAP, MyD88, and TRAF6 gene expression. HD11 cells were infected with vvIBDV (1×10^10^ copies/1×10^6^ cells) (A) or incubated with VLP (100 μg/mL) (B) or transfected with the recombinant plasmid expressing HA-VP2 (3 μg/1×10^6^ cells) (C), and levels of TIRAP, MyD88, and TRAF6 mRNA were assessed by RT-qPCR. (D, E) RT-qPCR (D) and immunoblot (E) analysis of TIRAP in HD11 cells untreated or treated with indicated shRNA (1 μg/1×10^5^ cells). Twenty-four hours later, cells were incubated with LPS (1 μg/mL) for 6 h. (F) Effect of TIRAP interference on IL-1β production. HD11 cells mock treated or infected with vvIBDV (1×10^10^ copies/1×10^6^ cells) in the presence or absence of shRNA-mediated specific gene silence pre-treated for 24 h and then harvested at 6 hpi, or stimulated with LPS (1 μg/mL) or VLP (100 μg/mL) in the presence or absence of shRNA-mediated specific gene silence pre-treated for 24 h and then harvested at 6 h. Total cell protein extracts were detected by immunoblot analysis, and protein production of IL-1β were measured by ELISA. (G, H) RT-qPCR (G) and immunoblot (H) analysis of MyD88 in HD11 cells untreated or treated with indicated shRNA (1 μg/1×10^5^ cells). Twenty-four hours later, cells were incubated with LPS (1 μg/mL) for 6 h. (I) Effect of MyD88 interference on IL-1β production. HD11 cells mock treated or infected with vvIBDV (1×10^10^ copies/1×10^6^ cells) in the presence or absence of shRNA-mediated specific gene silence pre-treated for 24 h were harvested at 6 hpi, or stimulated with LPS (1 μg/mL) or VLP (100 μg/mL) in the presence or absence of shRNA-mediated specific gene silence pre-treated for 24 h and then harvested at 6 h post stimulation. Total cell protein extracts were detected by immunoblot analysis, and IL-1β in the cell supernatants were measured by ELISA. (J) Effect of MyD88 inhibitor on IL-1β production. HD11 cells mock treated or pre-treated with ST2825 (15 μM) for 6 h were mock infected, or infected with vvIBDV (1×10^10^ copies/1×10^6^ cells) in the presence or absence of drug pre-treated for 6 h and then harvested at 6 hpi, or stimulated with LPS (1 μg/mL) or VLP (100 μg/mL) in the presence or absence of drug pre-treated for 6 h and then harvested at 6 h post stimulation. Total cell protein extracts were detected by immunoblot analysis, and protein production of IL-1β were measured by ELISA. (K, L) RT-qPCR (K) and immunoblot (L) analysis of TRAF6 in HD11 cells untreated or treated with indicated shRNA (1 μg/1×10^5^ cells). Twenty-four hours later, cells were incubated with LPS (1 μg/mL) for 6 h. (M) Effect of TRAF6 interference on IL-1β production. HD11 cells mock treated or infected with vvIBDV (1×10^10^ copies/1×10^6^ cells) in the presence or absence of shRNA-mediated specific gene silence pre-treated for 24 h were harvested at 6 hpi, or stimulated with LPS (1 μg/mL) or VLP (100 μg/mL) in the presence or absence of shRNA-mediated specific gene silence pre-treated for 24 h and then harvested at 6 h post stimulation. Total cell protein extracts were detected by immunoblot analysis, and IL-1β in the cell supernatants were measured by ELISA. (N) Luciferase assay of NF-κB promoter. HD11 cells co-transfected with NF-κB reporter plasmid (1 μg/1×10^6^ cells) and HA-TIRAP (1 μg/1×10^6^ cells), or HA-MyD88 (1 μg/1×10^6^ cells), or HA-TRAF6 (1 μg/1×10^6^ cells), and then harvested at 24 h post transfection. The protein production of Luciferase was measured by ELISA. HD11 cells stimulated with LPS (1 μg/mL) as a control. All data are representative of at least three independent experiments. Graphs show mean ± SD, n = 3, ^*^ P < 0.05, ** P < 0.01, *** P < 0.001.

TIRAP, MyD88, and TRAF6 are essential adaptors upstream of the NF-κB signaling pathway [45]. We further elucidate the mechanism by which IBDV regulates TIRAP, MyD88, and TRAF6. HD11 cells were infected with IBDV or treated with VLP or transfected with plasmids expressing HA-TIRAP, HA-MyD88, or HA-TRAF6, and the mRNA expression was monitored by RT-qPCR. The results showed that MyD88 had the most significant increase in mRNA levels in IBDV-infected (Fig 4A), VLP-treated (Fig 4B), or plasmids-transfected (Fig 4C) HD11 cells. In addition, the shRNA was designed to silence TIRAP expression in HD11 cells and its impact on IL-1β production was investigated. Firstly, with RT-qPCR (Fig 4D) and immunoblot (Fig 4E) experiments, three shRNA targeting TIRAP was screened and the interference efficiency of shTIRAP#2 (knockdown ≥ 60%) in HD11 cells during IBDV infection was the best. With the silence of TIRAP using shTIRAP#2, it was observed that the production of IL-1β significantly decreased in HD11 cells when compared with that in control cells infected with IBDV or stimulated with LPS and VLP (Fig 4F). At the same time, the shRNA was designed to silence MyD88 expression in HD11 cells and its impact on IL-1β production was investigated. The interference efficiency of shMyD88#1 (knockdown ≥ 60%) in HD11 cells during IBDV infection was the best (Fig 4G and 4H). With the silence of MyD88 using shMyD88#1, it was observed that the production of IL-1β significantly decreased in HD11 cells when compared with that in control cells infected with IBDV or stimulated with LPS and VLP (Fig 4I). The ST2825 (MyD88 inhibitor) was used to explore whether MyD88 affected the NF-κB-driven inflammatory response activated by IBDV. HD11 cells were IBDV-infected or LPS-stimulated or VLP-treated following ST2825 incubation and the IL-1β production was monitored by immunoblot and ELISA analyses. We observed that the production of IL-1β was significantly reduced (~50%) with ST2825 incubation in IBDV-infected or LPS-stimulated or VLP-treated HD11 cells (Fig 4J). Meanwhile, after being screened, the shTRAF6#2 (knockdown ≥ 55%) targeting TRAF6 was used to silence TRAF6 expression in HD11 cells ((Fig 4K and 4L). Immunoblot and ELISA results showed that IL-1β production in HD11 cells induced by IBDV or LPS or VLP was significantly down-regulated because of TRAF6 knockdown (Fig 4M). To investigate whether TIRAP, MyD88, and TRAF6 affects NF-κB-driven inflammatory response, we detected the effect of TIRAP, MyD88, and TRAF6 on NF-κB promoter activation in HD11 cells. The results revealed that MyD88 induced a more significant regulation of NF-κB activation than the other transfected samples (Fig 4N). Overall, MyD88 is deeply involved in the inflammatory response triggered by IBDV.

### The interaction of IBDV VP2 and MyD88 mediates the NF-κB signaling pathway activation

**Fig 5:**
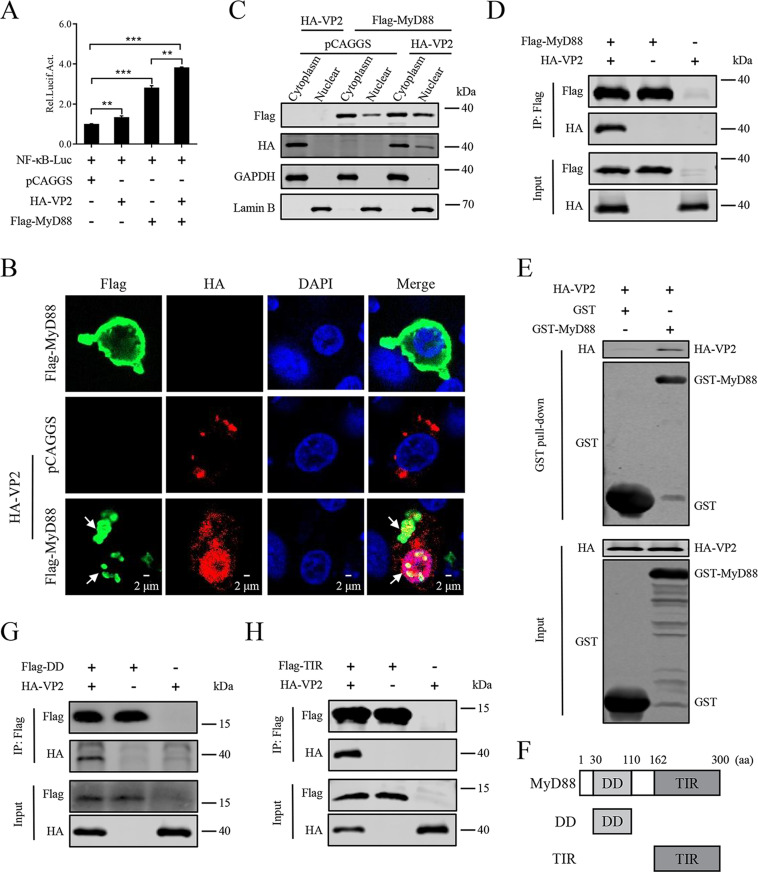
IBDV VP2 interacts with MyD88. (A) Luciferase assay of NF-κB promoter. DF-1 cells co-transfected with NF-κB reporter plasmid (1 μg/1×10^6^ cells) and HA-VP2 (1 μg/1×10^6^ cells) in the presence or absence of Flag-MyD88 (1 μg/1×10^6^ cells) and then harvested at 24 h post-transfection. The protein production of Luciferase was measured by ELISA. (B, C) Co-localization of MyD88 with VP2. HD11 cells transfected with the plasmid expressing Flag-MyD88 (2 μg/1×10^6^ cells) and HA-VP2 (2 μg/1×10^6^ cells) respectively or simultaneously for 24 h, MyD88 and VP2 were stained using the Flag Tag and HA Tag, respectively. The aggregation of MyD88 was highlighted with the white arrows (B). The cytoplasmic and nuclear extracts were detected by immunoblot analysis (C). (D) The interaction of VP2 and MyD88 detected by Co-IP. HEK293T cells were transfected with indicated plasmid (2 μg/1×10^6^ cells) and harvested at 30 h post-transfection. Cell lysates were immunoprecipitated using Flag Tag and analysed using the HA Tag and Flag Tag. (E) The interaction of VP2 and MyD88 detected by GST pull-down assay. Purified GST-MyD88 or GST was incubated with cell lysates of the plasmid expressing HA-VP2 (2 μg/1×10^6^ cells) in HEK293T cells. Cell extracts were incubated with GST agarose beads for 12 h. Mixtures were analysed by immunoblot using the HA Tag or GST Tag. (F) Schematic of the plasmids expressing the full-length MyD88 and its truncations (DD or TIR). (G, H) VP2 interacts with MyD88 truncations. HEK293T cells were transfected with indicated plasmid (2 μg/1×10^6^ cells) and harvested at 30 h post-transfection. Cell lysates were immunoprecipitated using the Flag Tag and analyzed using the HA and Flag Tag. All data are representative of at least three independent experiments. Graphs show mean ± SD, n = 3, ** P < 0.01, *** P < 0.001.

To further explore the mechanism by which VP2 regulates MyD88, we examined the effect of VP2 on MyD88-driven NF-κB promoter activation in HD11 cells. The results revealed that the VP2 and MyD88 induced positive regulation of NF-κB activation was more significant than in the mock-transfected sample (Fig 5A). To verify how VP2 regulates MyD88, HD11 cells were transfected with the Flag-MyD88 plasmid or co-transfected with the HA-VP2 plasmid and either the Flag-MyD88 or pCAGGS plasmid. The results of confocal microscopy experiments showed that MyD88 mainly concentrated in the cytoplasm adjacent to the cytomembrane. However, MyD88 exhibited significant cluster-like accumulation in the cytoplasm, and some MyD88 entered the nucleus when co-transfected with the plasmid expressing VP2 in HD11 cells, whereas VP2 co-localized with MyD88 in both the cytoplasm and nucleus (Fig 5B and 5C).

To further identify the relation of VP2 and MyD88, the co-immunoprecipitation (Co-IP) was performed in HEK293T cells. The results indicated that VP2 interacted with MyD88 (Fig 5D), which was further confirmed by the Glutathione S-transferase (GST) pull-down assay (Fig 5E). As MyD88 contains a death domain (DD) (amino acids [aa] 30 to 110), an intermediate (INT) domain (aa 111 to 162), and a toll/interleukin-1 receptor (TIR) domain (aa 163 to 300), to further map the key elements of MyD88 interacting with VP2, the plasmid expressing DD (aa 30 to 110) or TIR (aa 163 to 300) truncation of MyD88 was constructed (Fig 5F) and its interaction with VP2 was analysed. The Co-IP results demonstrated that both the DD and TIR domains of MyD88 interact with VP2 (Fig 5G and 5H). These results indicate that VP2 interacts with MyD88.

### IBDV VP2 enhances MyD88 complex assembly and MyD88 oligomerization

**Fig 6:**
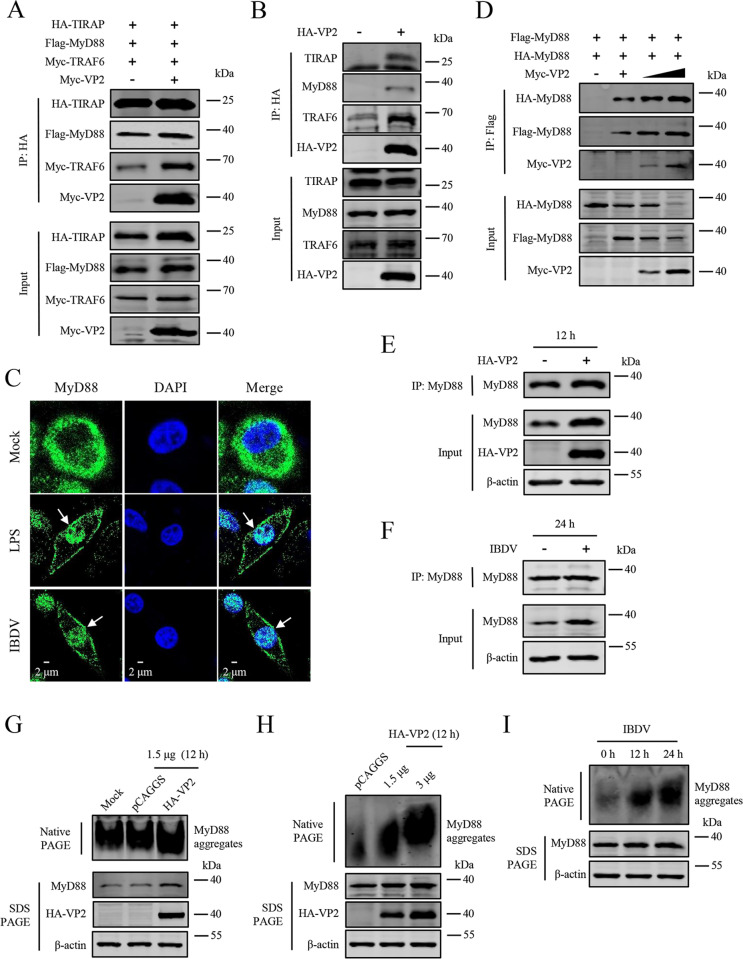
IBDV VP2 enhances MyD88 complex assembly and MyD88 oligomerization. (A) VP2 promotes the associations of TIRAP-MyD88-TRAF6. HD11 cells were co-transfected with indicated plasmids (1 μg/1×10^6^ cells) for 24 h. Cell lysates were immunoprecipitated using the HA Tag and analysed using the HA, Flag, and Myc Tag. (B) VP2 promotes the association of endogenous TIRAP-MyD88-TRAF6. HD11 cells were transfected with the plasmid expressing HA-VP2 (3 μg/1×10^6^ cells) for 12 h. Cell lysates were immunoprecipitated using the HA Tag and analysed using TIRAP, MyD88, TRAF6 monoclonal antibody, and HA Tag. (C) Effect of IBDV on MyD88 distribution. HD11 cells were infected with vvIBDV (1×10^10^ copies/1×10^6^ cells) or stimulated with LPS (1 μg/mL) for 12 h, and MyD88 was stained using MyD88 monoclonal antibody. The aggregation of MyD88 was highlighted with the white arrows. (D) VP2 enhances the oligomerization of MyD88. HD11 cells were transfected with plasmids expressing Flag-MyD88 (1.5 μg/1×10^6^ cells), HA-MyD88 (1.5 μg/1×10^6^ cells), and increasing doses of Myc-VP2 (0, 0.7, 1.5 μg) for 24 h. Cell lysates were immunoprecipitated using the Flag Tag and analysed using the HA, Flag, and Myc Tag. (E) VP2 enhances the oligomerization of endogenous MyD88. HD11 cells were transfected with plasmid expressing HA-VP2 (3 μg/1×10^6^ cells) for 12 h. Cell lysates were immunoprecipitated using HA Tag and analysed using MyD88 and HA Tag antibodies. (F) IBDV enhances the oligomerization of endogenous MyD88. HD11 cells were infected with vvIBDV (1×10^10^ copies/1×10^6^ cells) for 24 h. Cell lysates were immunoprecipitated and analysed using anti-MyD88 antibody. (G-H) VP2 enhances oligomerization of MyD88 in the natural condition in HD11 cells. HD11 cells were transfected with the plasmid expressing HA-VP2 (3 μg/1×10^6^ cells) for 12 h. Cell lysates were separated by native or SDS PAGE and analysed by immunoblot with the indicated antibodies. (I) IBDV enhances oligomerization of MyD88 in the natural condition in HD11 cells. HD11 cells were infected with vvIBDV (1×10^10^ copies/1×10^6^ cells) for 12 and 24 h. Cell lysates were separated by native or SDS PAGE and analysed by immunoblot with the indicated antibodies. HD11 cells stimulated with LPS (1 μg/mL) as a control. All data are representative of at least three independent experiments.

MyD88 is the core of myddosome, in which the TIR domain of MyD88 functionally interacts with TIRAP. The activation of myddosome drives the subsequent recruitment of TRAF6, which further activates the NF-κB to mediate the inflammatory responses [46]. Since the activation of MyD88 is associated with TIRAP and TRAF6, to further verify whether viral VP2 is involved in the assembly of the MyD88 complex, HD11 cells were co-transfected with plasmids expressing HA-TIRAP, Flag-MyD88, Myc-TRAF6, and either Myc-VP2 or pCAGGS. Co-IP experiments results showed that VP2 significantly promoted interaction among TIRAP, MyD88, and TRAF6 (Fig 6A). To further elucidate whether VP2 strengthens the interaction of endogenous MyD88 complex, we performed Co-IP experiments using antibodies against endogenous TIRAP, MyD88, and TRAF6 in HD11 cells. The results showed that VP2 overexpression enhanced the endogenous MyD88 complex assembly (Fig 6B).

Previous studies have demonstrated that a large number of oligomers formation is representative of MyD88 activation, the crucial step of macromolecular myddosome complex activation is the assembly of six helical oligomers of MyD88 which have the enzymatic activity, called “MyD88 oligomerization” [47]. MyD88 biochemically interacts with TLR/IL-1R through the heterotrimeric TIR domain, which has no endogenous enzymatic activity. Once the ligand binding, TLR/IL-1R associated receptor dimerization, the DD domain of MyD88 couples to enzymatic activity and self-stacks consisting of six MyD88s [48,49]. In the preceding studies, the formation of cluster-like structures may suggest that the MyD88 oligomerization in HD11 cells (Fig 5B). Accordingly, in confocal microscopy experiments, HD11 cells were infected with IBDV or stimulated with LPS, and endogenous MyD88 dispersed through the cytoplasm under normal physiological conditions, whereas MyD88 aggregated around the cytomembrane or entered the nucleus during IBDV infection or LPS simulation (Fig 6C). To further clarify the effect of VP2 on MyD88 oligomerization, plasmids expressing Flag-MyD88 and HA-MyD88 with either Myc-VP2 or pCAGGS were co-transfected in HD11 cells. The Co-IP results revealed that VP2 markedly enhanced the interaction between Flag-MyD88 and HA-MyD88 in a dose-dependent manner (Fig 6D). To further determine whether endogenous MyD88 oligomerization could be affected by VP2, we conducted further Co-IP experiments in HD11 cells with plasmid expressing HA-VP2 for 12 h (Fig 6E) or IBDV infection for 24 h (Fig 6F) and discovered that the oligomerization of endogenous MyD88 was significantly increased. As described earlier, the self-oligomerization of MyD88 is concomitant with gradual formation of multimers, which have a remarkably high molecular weight in natural state [48,49]. Moreover, we performed native PAGE experiments using an antibody against MyD88 in HD11 cells to verify the aggregation of MyD88 with a natural protein structure. Similarly, we found that the endogenous MyD88 oligomerization remarkably enhanced in a dose-dependent manner with VP2 overexpression (Fig 6G and 6H) and in a time-dependent manner with IBDV infection (Fig 6I).

### Residues 253 and 284 of VP2 are involved in IBDV-induced inflammatory response

**Fig 7:**
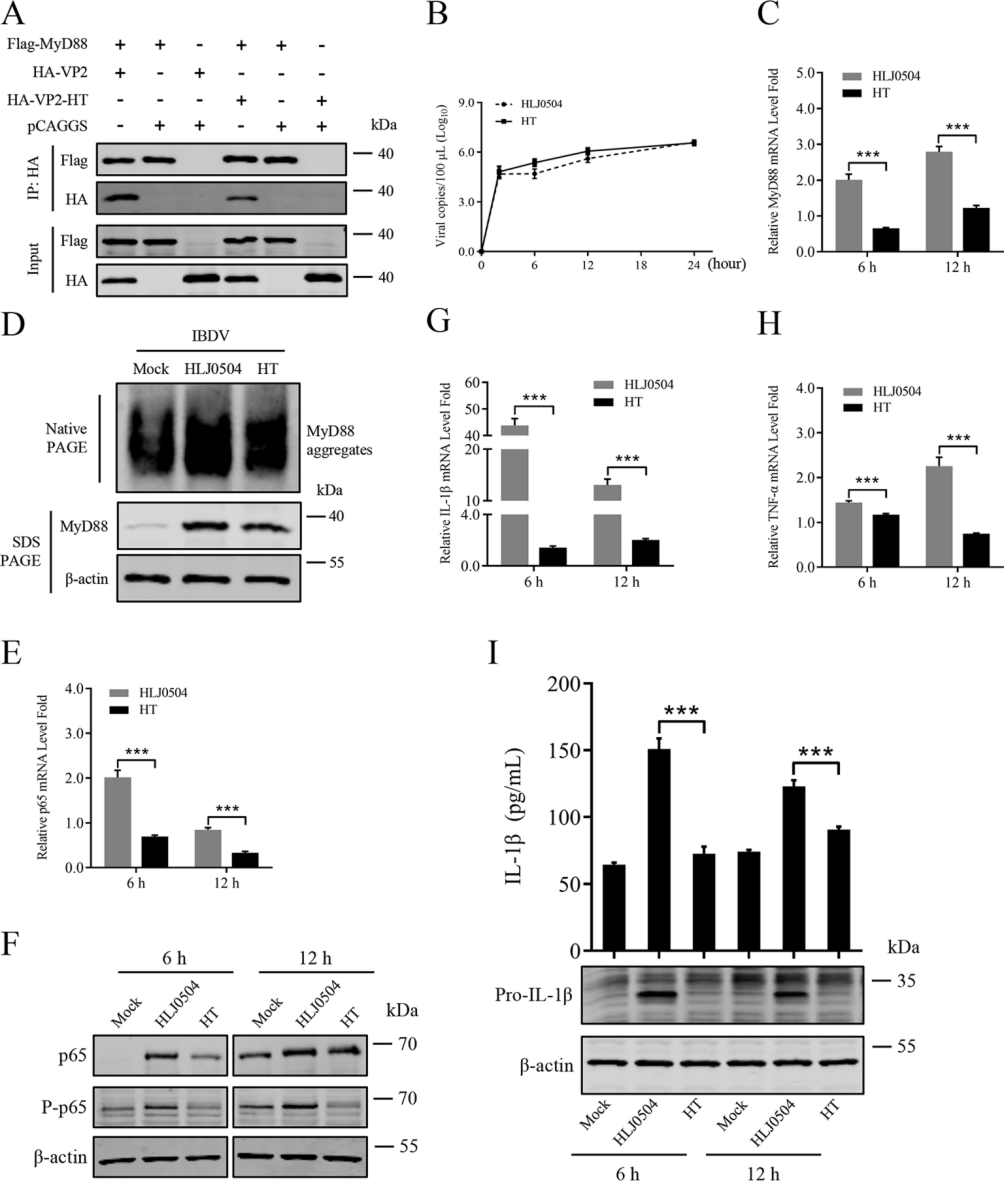
Residues 253 and 284 of VP2 are involved in the difference of inflammatory response induced by different IBDV strains. (A) Residues 253/284 mutations downregulate the interaction of VP2 and MyD88. HEK293T cells were transfected with indicated plasmids (2 μg/1×10^6^ cells) for 30 h. Cell lysates were immunoprecipitated using Flag Tag and analysed using the HA Tag and Flag Tag. (B) The growth dynamics of HLJ0504 or HT strain of IBDV in HD11 cells were analysed by RT-qPCR. (C, D) Effect of HLJ0504 or HT strain on MyD88 gene expression and production. HD11 cells were infected with HLJ0504 or HT strain (1×10^10^ copies/1×10^6^ cells), and levels of MyD88 mRNA were assessed by RT-qPCR at 6 and 12 hpi (C), and total cell protein extracts were analysed by native or SDS PAGE and probed with specific antibodies shown on the left (D). (E, F) Effect of HLJ0504 or HT strains on NF-κB mediated gene expression and production. HD11 cells were infected with HLJ0504 or HT (1×10^10^ copies/1×10^6^ cells) for 6 and 12 hpi, and levels of p65 mRNA were assessed by RT-qPCR (E), and total cell protein extracts were analysed by immunoblot analysis, and probed with specific antibodies shown on the left (F). (G-I) Effect of HLJ0504 or HT strain on the expression of IL-1β, TNF-α, and production of IL-1β. HD11 cells were infected with HLJ0504 (1×10^10^ copies/1×10^6^ cells) or HT strain (1×10^10^ copies/1×10^6^ cells) for 6 and 12 hpi, and levels of IL-1β (G) and TNF-α (H) mRNA were assessed by RT-qPCR. The pro-IL-1β in the cell lysates were analysed by immunoblot analysis, and IL-1β in the cell supernatants were measured by ELISA (I). All data are representative of at least three independent experiments. Graphs show mean ± SD, n = 3, *** P < 0.001.

Based on our previous studies, residues 253 and 284 of VP2 significantly affect the virulence of IBDV [50], However whether these two residues are involved in the IBDV-induced inflammatory response has never been reported. Considering that the interaction of VP2 with MyD88 mediates the NF-κB-driven IBDV-induced inflammatory response, we first examined whether they affect the interaction of VP2 and MyD88. The plasmid expressing VP2-HT with double mutation Q253H/ A284T was constructed. Co-IP results showed that both VP2 and VP2-HT interact with MyD88, whereas VP2-HT showed a decreased (~2.9-fold) interaction capacity compared to VP2 (Fig 7A).

Furthermore, the growth kinetics of the vvIBDV HLJ0504 strain and medium-virulence HT strain were compared in HD11 cells. IBDV strains HLJ0504 and HT infected HD11 cells with similar growth kinetics in the early stages of viral infection (Fig 7B). Additionally, RT-qPCR experiments showed that the mRNA levels of MyD88 in HT-infected HD11cells were significantly lower than that in the HLJ0504-group at 6 and 12 hpi (Fig 7C), and native PAGE experiments showed that both HLJ0504 and HT strains could up-regulated the aggregation of endogenous MyD88 in HD11 cells, with the HT strain group being comparatively lower (Fig 7D). Besides, HD11 cells were inoculated with HLJ0504 and HT strains for 6 and 12 h, and the p65 expression was monitored by RT-qPCR (Fig 7E) and immunoblot (Fig 7F) analysis, which showed that HLJ0504 increased the expression and phosphorylation levels of p65, but this effect was much decreased in the HT strain-infected HD11 cells.

We next investigated whether HT promotes the release of pro-inflammatory cytokines IL-1β and TNF-α in HD11 cells. RT-qPCR results showed that HT strain triggered relatively lower transcription of IL-1β (Fig 7G) and TNF-α (Fig 7H) in HD11 cells compared to HLJ0504 group. Moreover, the protein levels of IL-1β in cell lysates and cell supernatants were detected by immunoblot and ELISA, respectively. Additionally, we demonstrated that residues 253 and 284 of VP2 are crucial for IBDV-induced inflammation in DT40 cells (S1 Fig).

These results revealed that both HLJ0504 and HT strains were able to elevate IL-1β production, while the ability of HT was extremely weak when compared to HLJ0504 (Fig 7I). These results indicated that residues 253 and 284 of VP2 are dramatically involved in the IBDV-induced inflammatory response.

## Discussion

In the long-term confrontation with the organism, IBDV has evolved various approaches to escape the host immune system. Recently, there have been elucidated that IBDV could impair the ability of transcriptional regulatory factors translocate into the nucleus, leading to the inhibition of IFN and pro-inflammatory cytokines expression [51]. The inflammatory response is an essential component of innate immunity to defense pathogens [12–14]. Based on our results, we demonstrate that the NF-κB signaling pathway is activated by the interaction of viral VP2 and MyD88 and the resulting pro-inflammatory cytokines including IL-1β are produced during IBDV infection, which is the first to reveal the molecular mechanism by which IBDV triggers inflammation.

The major hallmarks of IBDV infection are rapid progression and intense inflammatory response. Uncontrolled inflammatory response is thought to be the main reason for disease exacerbation [29,30]. There has been reported that vvIBDV induced strong pro-inflammatory responses in bursal tissue by increasing IL-1β, IL-6, and CXCLi2 mRNA transcription [29]. IBDV infection in two chicken lines leads to severe depletion of B cells and more-extensive p53-related induction in the bursa, inducing a faster inflammatory response [30]. In this study, several evidences demonstrate that IBDV infection induces severe inflammation of chickens. For infected chickens, the inflammatory factors including IL-1β and TNF-α in serum increased; the bursa was severely damaged with obvious hemorrhage and inflammatory mucus secretion; the bursa lymphoid follicles were atrophied with massive lymphocytes depletion and inflammatory cells infiltration including macrophages.

Macrophages may play an important role in the infection and inflammation process of IBDV. It is thought that the invading IBDV infects intestinal macrophages, after which IBDV is carried into bursa-beginning proliferation as the macrophages migration. It has been reported that the chicken macrophage migration inhibitory factor (chMIF) secreted by primary bursal cells could induce the migration of peripheral blood mononuclear cells and promote the transcription of pro-inflammatory cytokines in chicken macrophages upon IBDV infection [52].

In this study, the chicken macrophage cell line, HD11, was used to investigate the association between IBDV infection and inflammation. Two hours after virus inoculation, IBDV particles were engulfed by the pseudopodia of the HD11 cells and entered the cytoplasm. Macrophages participate in the inflammatory response mainly by secreting various cytokines, including IL-1β. The production of biologically activated IL-1β with biological activity usually involves two events. Initially, host defenses recognize pathogens invasion and activate downstream correlated signaling pathways, such as the NF-κB signaling pathway. This induces the synthesis of pro-IL-1β, which was an inactive precursor. Whereafter, post-translational modifications mediated by inflammasome activation catalyze pro-IL-1β maturation and secretion, turning pro-IL-1β into IL-1β. Based on our results, in IBDV-infected HD11 cells for 6-24 hpi, the up-regulation of pro-IL-1β and IL-1β were detected in cell lysate and cell supernatant, respectively, which indicates that IBDV infection promotes inflammation response in HD11 cells.

Previous studies have found that the viral capsid protein, as a strong stimulus, can be recognized by PRRs to induce inflammatory response [12–14]. As the only capsid protein in IBDV, VP2 is located on the outermost surface of the viral particle and may play an important role in triggering the inflammatory response. To verify this speculation, the VLP composed of VP2 was used to incubate with HD11 cells. Results showed that VLP was phagocytosed into HD11 cells and the expression and secretion of IL-1β was up-regulated. IBDV has completed virus replication within 24 h. Based on our results, in VP2-transfected HD11 cells for 6-24 hpi, the up-regulation of pro-IL-1β and IL-1β were detected in cell lysate and cell supernatant, respectively, which indicates that both the VP2 from input virus and newly synthesized VP2 in the cell could trigger inflammation response in HD11 cells. These results indicate that the capsid protein VP2 of IBDV is an important inflammatory stimulus.

The NF-κB signaling pathway mediates the transcription of pro-IL-1β, which consequently triggers inflammation response. Nuclear translocation of p65 subunit in NF-κB is a symbol of NF-κB signaling pathway activation [40,41,43]. To explore the mechanism by which IBDV activates the NF-κB signaling pathway, HD11 cells were inoculated with IBDV or transfected with plasmid expressing viral VP2 for 6-24 h. Our results demonstrate that both IBDV infection and VP2 overexpression increase the gene expression, production, phosphorylation, and nuclear translocation of p65, leading to the activation of NF-κB signaling pathway and downstream signal transduction associating with inflammatory response.

MyD88 is the core of the myddosome, which is an essential adaptor responsible for transmitting signals from PRRs to NF-κB signaling pathway in inflammatory response [11]. Our results showed that, in the presence of IBDV VP2, MyD88 was up-regulated and beneficial to NF-κB activation. To study how viral VP2 regulates MyD88, the relationship of VP2 and MyD88 was further identified. Results of confocal microscopy, Co-IP, and GST pull-down confirmed the interaction of VP2 and MyD88. The interaction of VP2 and MyD88 altered the subcellular localization of MyD88. MyD88 mainly concentrated in the cytoplasm near the cytomembrane, but exhibited significantly cluster-like accumulation in the cytoplasm and some entered the nucleus when co-expressed with viral VP2. Similarly, a portion of MyD88 translocated from the cytoplasm to the nucleus upon stimulation with LPS or IBDV. There have been reported that the nuclear translocation of MyD88 is associated with the regulation of TLR-related signaling pathways [53–56]. IBDV VP2 interacts with both the DD and TIR domains of MyD88. However, it has been reported that the peste des petits ruminants virus (PPRV) N protein only interacted with TIR domain of MyD88 [57]. Different viruses trigger MyD88-activated response in different ways. These results provide a deeper understanding about oligomerization of MyD88 during virus infection. Of course, the details of MyD88 oligomerization, MyD88 complex assembly, and its signal transduction in IBDV induced inflammation deserve further in-depth study.

When compared to the vvIBDV HLJ0504 strain with a high mortality, the medium virulence IBDV strain with Q253H/ A284T mutations of VP2 has no lethality and induces lower inflammatory response [50,51]. Some amino acid residues are critical for proteins to perform functions and affect the process of the inflammatory response [58,59]. Our study shows that residues 253/284 mutations of viral VP2 not only attenuate the strength of the interaction between VP2 and MyD88, but significantly reduce the expression and production of MyD88, p65, and IL-1β in the axis of MyD88-NF-κB-IL-1β signaling pathway, which greatly diminish the induced inflammation. Thus, residues 253/284 of VP2 are deeply involved in IBDV-induced inflammatory response. Our previous results have proven that low-dose (1×10^3^ copies/1×10^6^ cells-1×10^9^ copies/1×10^6^ cells) HT infects HD11 cells could not activate the inflammatory response. In order to better compare the differences in activating the inflammatory response between vvIBDV and HT strains, we observed weak activation of the inflammatory response only when the dose was increased to 1×10^10^ copies/1×10^6^ cells, which is why we finally chose 1×10^10^ copies/1×10^6^ cells as the infection dose of IBDV.

Collectively, to our knowledge, this study was the first to demonstrate that IBDV capsid protein VP2 is an important inflammatory stimulant and revealed its molecular mechanism associated with MyD88 oligomerization. Certainly, viral infection first activates PRR(s) located on the cell surface, and then transmits signals to MyD88 through ligand binding, which is also an important mechanism for virus triggered inflammation [3,4]. In other words, the enhancement of MyD88 oligomerization may not only be due to direct stimulation but also due to stimulation through TLRs and other PRRs. About ten TLRs of chicken have been identified, which possess several unique characteristics in terms of ligand specificity, the formation of TLR receptor complexes, and the activation of TLR signaling pathways [60]. In this study, no direct data was presented to exclude roles for TLRs and other PRRs in activating inflammation following IBDV infection. The more comprehensive and detailed mechanism of the inflammatory process triggered by IBDV deserves further exploration. Overall, we discovered that IBDV infection induces high levels of IL-1β and the viral VP2 plays critical role in promoting the production of IL-1β. Mechanically, VP2 binds to MyD88 to enhance MyD88 oligomerization and promote MyD88 complex assembly, subsequently, facilitates nuclear translocation of p65, which in consequence increases IL-1β production. Our findings reveal one key molecular mechanism by which IBDV triggers inflammation.

## Materials and methods

### Ethics statement

All the chickens were purchased from the National Poultry Experimental Animal Resource Bank of the HVRI and housed in a negative pressure isolator at the Experimental Animal Center of the HVRI. The animal experiment was conducted in compliance with the Animal Welfare Act and Guide for the Care and Use of Laboratory Animals, approved by the Laboratory Animal WelfareCommittee of Harbin Veterinary Research Institute (HVRI) of the Chinese Academy of Agriultural Sciences (approval number 230724-01-GR).

### Cells and viruses

Chicken fibroblast cells DF-1 and Human embryonic kidney (HEK293T) cells were cultured in Dulbecco’s modified minimal essential medium (DMEM) (Sigma-Aldrich) supplemented with 10% fetal bovine serum (FBS) (Sigma-Aldrich), 100 U/ml penicillin, and 100 mg/ml streptomycin. Chicken macrophage cells HD11 were cultured in RPMI1640 medium (Sigma-Aldrich) complemented with 10% FBS (Sigma-Aldrich), 2% chicken serum (Sigma-Aldrich), 1% sodium pyruvate (Sigma-Aldrich), 1% non-essential amino acids (Gibco), and 1‰ β-mercaptoethanol (Sigma-Aldrich). Except for HD11 cells (38.5°C), other cells were cultured in a humidified incubator at 37°C with 5% CO_2_.

The representative epidemic strain of very virulent IBDV (vvIBDV) named HLJ0504 (GenBank No: GQ451330/GQ451331) [61] was isolated and identified by the Avian Immunosuppressive Disease Division at Harbin Veterinary Research Institute (HVRI), the Chinese Academy of Agricultural Sciences (CAAS) (referred to as “our laboratory” in this paper). The rescued IBDV strain (referred to as the HT strain, GenBank No: PQ773517/PQ773518) [48,51] from the parent backbone HLJ0504 strain with double mutations of Q253H/A284T in VP2 was preserved in our laboratory. Compared to the vvIBDV HLJ0504 strain with high mortality, HT is a medium virulent strain and is not lethal.

### Plasmids

Luciferase reporter plasmid (NF-κB-Luc) was purchased from Beyotime Biotechnology. The VP2 gene of the IBDV strains HLJ0504 or HT was cloned into the pCAGGS vector, with an HA, Myc or Flag tag fused to the N-terminus. Plasmids encoding chicken p65 (GenBank accession No. NM_001396038.1), TIRAP (GenBank accession No. NM_001024829.1), MyD88 (GenBank accession No. NM_001030962.5), or TRAF6 (GenBank accession No. XM_046942060.1) were constructed by cloning the synthesized sequences into pCAGGS vector containing Myc, HA, or Flag tags. The recombinant plasmid pGEX-GST-MyD88 was constructed by cloning the chicken *MyD88* gene (GenBank accession No. NM_001030962.5) into the pGEX-4T-1 vector with a GST tag fused to the N-terminus.

### Antibodies and reagents

The mouse anti-HA monoclonal antibody (mAb, H9658), rabbit anti-HA polyclonal antibody (pAb, H6908), mouse anti-Flag mAb (F1804), rabbit anti-Flag pAb (SAB4301135), mouse anti-Myc mAb (M4439), rabbit anti-Myc pAb (C3956), rabbit anti-GST pAb (G7781), mouse anti-β-actin mAb (A5441) were purchased from Sigma-Aldrich. Rabbit anti-pro-IL-1β pAb, Rabbit anti-p65 pAb, rabbit anti-p-p65 pAb, rabbit anti-TIRAP pAb, rabbit anti-MyD88 pAb, rabbit anti-TRAF6 pAb were developed by immunizing the rabbits with recombinant proteins (Biodragon). Rabbit anti-IκBα pAb (T55026) and rabbit anti-p-IκBα pAb (T55572) were purchased from Abmart. Rabbit anti-Lamin B1 (ab229025) was purchased from Abcam. Rabbit anti-GAPDH (AC001) was purchased from ABclonal. IRDye 800CW goat anti-mouse (926-32210) and IRDye 680LT goat anti-rabbit (926-68021) secondary antibodies were purchased from LiCor Bio-Sciences. Mouse anti-VP2 mAb of IBDV was produced and preserved in our laboratory. LPS (HY-D105), BAY 11-7082 (NF-kB inhibitor, HY-13453), ST2825 (MyD88 inhibitor, HY-50937) were purchased from MedChem Express.

### Preparation of viral-like particle (VLP)

The VP2 gene of the vvIBDV HLJ0504 strain with a His-tag at the N-terminus was subcloned into the prokaryotic expression plasmid pCold Ⅰ (Takara). The recombinant plasmid was transformed into the engineered *Escherichia coli* (*E. coli*) BL21 (DE3) (Takara) to express the recombinant VP2. Recombinant VP2 was further purified via ammonium sulfate precipitation, followed by size-exclusion chromatography. Transmission electron microscopy revealed that the purified protein could self-assembled into 25-nm VLP. The detailed methods are based on our previous publication [62].

### Animal experiments

Three-week-old specific-pathogen-free (SPF) chickens were randomly allocated into two experimental groups. One group (45 chickens) was infected with the vvIBDV HLJ0504 strain at the dose of 36 copies per chicken via the ocular and intranasal routes. The other group (20 chickens) was mock-infected and served as a control. Clinical symptoms were monitored daily. At 6 h, 12 h, 1 d, 3 d, 5 d, and 7 d post-infection, seven infected and three mock chickens per group were humanely sacrificed and their serum and bursa of Fabricius (bursa) were harvested. The serums were used to assess cytokines levels (for IL-1β and TNF-α) with chicken cytokines ELISA kit (Cloud-Clone). The bursa were fixed in 10% buffered formalin, embedded in paraffin, sectioned, and stained with hematoxylin and eosin analysis.

### Virus growth kinetics

The multi-step viral growth kinetics of HLJ0504 and the HT strain of IBDV in HD11 cells were analysed. The cells were infected with 1×10^10^ copies/1×10^6^ cells and collected at 2, 6, 12, and 24 hpi, and tested for viral genome copies using RT-qPCR assays.

### Co-immunoprecipitation (Co-IP) and immunoblot analysis

For Co-IP, cells transfected with the indicated plasmids were lysed in NP-40 lysis buffer (Beyotime) containing a protease inhibitor cocktail (Roche) and centrifuged at 13,000 g at 4˚C for 5 min. Whole cell lysate were precleared with protein A/G agarose and then incubated with anti-tag beads or the appropriate antibody and protein A/G agarose at 4˚C overnight with constant rotation. Co-IP samples were collected by centrifugation and washed with PBS ten times. After washing, the cellular lysate protein samples were boiled with 5×SDS loading buffer (Beyotime) for 10 min and subjected to immunoblot analysis.

For the immunoblot analyses, the protein lysates were separated by electrophoresis on SDS-PAGE gels and then the proteins were transferred onto a nitrocellulose membrane. The membrane was blocked with 5% (w/v) skim milk in PBST for 1 h and then incubated with the corresponding primary antibodies diluted in PBS. After washing with PBST, the membrane was incubated with the appropriate secondary antibodies diluted in PBS. Finally, the membrane was washed four times with PBST and scanned using an Odyssey Infrared Imaging System (Li-Cor Bio-sciences) for further analysis.

### RT-qPCR

Whole-cell RNA was extracted from transfected, infected, or uninfected mock cells at the indicated time points using the RNAiso Plus kit (9109, TaKaRa, Japan). The extracted RNA was reverse-transcribed into cDNA using HiScript II QRT SuperMix for quantitative PCR (qPCR) (R223-01, Vazyme, China). Each sample was triplicated for relative abundance analysis of individual mRNA transcripts from the viral genome or host factors, with the 28s mRNA serving as the normalizing reference. The RT-qPCR amplification reaction utilized the SYBR Green qPCR Kit (QPS-201, TOYOBO, Japan) with the following cycling conditions (host factors): initial denaturation at 95°C for 2 min, followed by 40 cycles of 95°C for 5 s, 60°C for 30 s, and a melt curve. The results were analysed using the 2-ΔΔC method. To determine the viral loads of IBDV in infected cells, the Premix Ex Taq (Probe qPCR; R390B, TaKaRa, Japan) was employed with cycling conditions of 48°C for 30 min and 95°C for 20 s, followed by 40 cycles of 95°C for 3 s, and 60°C for 30 s. The primers used in this study are available upon request.

Primers sequences are as follows: *IL-1β* (F: 5’-CCGAGGAGCAGGGACTTT-3’, R: 5’-AGGACTGTGAGCGGGTGTAG-3’), *TNF-α* (F: 5’-AGCAGGGCTGACACGGAT-3’, R: 5’-TGTTGGCATAGGCTGTCCTG-3’), *p65* (F: 5’-TGCGGTTCCGCTATAAGTGT-3’, R: 5’-CGGTAATGGTTTACGCGGATG-3’), *TIRAP* (F: 5’-TTCCTGTTATGGCCGGATGG-3’, R: 5’-TGAAAGTGAGTGGCTGGTGG-3’), *MyD88* (F: 5’-GGATGATCCGTATGGGCATGG-3’, R: 5’-CTCCGTTTGCTCCAACTCTCT-3’), *TRAF6* (F: 5’-GTGTCCAAGGCGTCAAGTCT-3’, R: 5’-GCAGGTTTGGTCATGAAGCTCT-3’).

### RNA interference

Control, chicken p65, and chicken MyD88 siRNAs were purchased from Sigma-Aldrich. HD11 cells were transfected with siRNAs (1 μg/1×10^5^ cells) using Lipofectamine 2000 (Invitrogen) following the manufactures’ protocol. At 24 h post-transfection, the cells were infected with IBDV (1×10^10^ copies/1×10^6^ cells). At 6 h post-infection, HD11 cells were examined using RT-qPCR or SDS-PAGE.

### Luciferase assays

DF-1 cells were transfected with luciferase reporter plasmid (NF-κB-Luc) and the indicated plasmids. At 24 h post-transfection, the cells were harvested, and luciferase activity was detected using a dual luciferase reporter kit (ThermoFisher Scientific) according to the manufacture’s protocol.

### Native polyacrylamide gel electrophoresis (native-PAGE)

HD11 cells were collected and washed twice with PBS. The cells were then lysed with native lysis buffer (ab156035, Abcam), supplemented with protease inhibitor cocktail (Roche) for 30 min on ice. The lysates were centrifuged at 13,000 g for 5 min and added 5× native loading buffer, and then subjected to immunoblot analysis.

### Glutathione S-transferase (GST) pull-down assays

The GST-MyD88 was purified by glutathione agarose beads (GE Healthcare). HA-VP2 was purified by using Ni-nitrilotriacetic acid agarose (GE Healthcare). The purified proteins were incubated with glutathione agarose beads for 6 h. The beads were washed three times with PBS, mixed with 5× SDS loading buffer and boiled for 10 min and then analysed by immunoblot analysis.

### Statistical analysis

All statistical analyses were performed using the GraphPad Prism software. Differences were considered statistically significant if the P value was less than 0.05.

## Supporting information

S1 Fig Residues 253 and 284 of VP2 are involved in the difference of inflammatory response induced by different IBDV strains in DT40 cells.
(A) The growth dynamics of the HLJ0504 and HT strain of IBDV in DT40 cells were analysed using RT-qPCR. (B-D) Effect of HLJ0504 or HT strain on the expression of IL-1β, TNF-α, and production of IL-1β. DT40 cells were infected with HLJ0504 or HT strain (1×10^10^ copies/1×10^6^ cells) for 6 and 12 hpi, and levels of IL-1β (B) and TNF-α (C) mRNA were assessed by RT-qPCR. The pro-IL-1β in the cell lysates were analysed by immunoblot analysis, and IL-1β in the cell supernatants were measured by ELISA (D). All data are representative of at least three independent experiments. Graphs show mean ± SD, n=3, ***, P<0.001.
(TIF)

S1 Data Excel spreadsheet containing, in separate sheets, the underlying numerical data and statistical analysis for Figures.
(XLSX)

## References

[1] Akira S. Pathogen recognition by innate immunity and its signaling. Proc Jpn Acad Ser B Phys Biol Sci. 2009;85(4):143–56. doi: 10.2183/pjab.85.143 19367086 PMC3524297

[2] Koyama S, Ishii KJ, Coban C, Akira S. Innate immune response to viral infection. Cytokine. 2008;43(3):336–41. doi: 10.1016/j.cyto.2008.07.009 18694646

[3] Kawai T, Akira S. The role of pattern-recognition receptors in innate immunity: update on Toll-like receptors. Nat Immunol. 2010;11(5):373–84. doi: 10.1038/ni.1863 20404851

[4] O’Neill LAJ, Golenbock D, Bowie AG. The history of Toll-like receptors - redefining innate immunity. Nat Rev Immunol. 2013;13(6):453–60. doi: 10.1038/nri3446 23681101

[5] Sims JE, Smith DE. The IL-1 family: regulators of immunity. Nat Rev Immunol. 2010;10(2):89–102. doi: 10.1038/nri2691 20081871

[6] Burns K, Martinon F, Esslinger C, Pahl H, Schneider P, Bodmer JL, et al. MyD88, an adapter protein involved in interleukin-1 signaling. J Biol Chem. 1998;273(20):12203–9. doi: 10.1074/jbc.273.20.12203 9575168

[7] Muzio M, Ni J, Feng P, Dixit VM. IRAK (Pelle) family member IRAK-2 and MyD88 as proximal mediators of IL-1 signaling. Science. 1997;278(5343):1612–5. doi: 10.1126/science.278.5343.1612 9374458

[8] Wesche H, Henzel WJ, Shillinglaw W, Li S, Cao Z. MyD88: an adapter that recruits IRAK to the IL-1 receptor complex. Immunity. 1997;7(6):837–47. doi: 10.1016/s1074-7613(00)80402-1 9430229

[9] Medzhitov R, Preston-Hurlburt P, Kopp E, Stadlen A, Chen C, Ghosh S, et al. MyD88 is an adaptor protein in the hToll/IL-1 receptor family signaling pathways. Mol Cell. 1998;2(2):253–8. doi: 10.1016/s1097-2765(00)80136-7 9734363

[10] Cao Z, Xiong J, Takeuchi M, Kurama T, Goeddel DV. TRAF6 is a signal transducer for interleukin-1. Nature. 1996;383(6599):443–6. doi: 10.1038/383443a0 8837778

[11] Janssens S, Burns K, Tschopp J, Beyaert R. Regulation of interleukin-1- and lipopolysaccharide-induced NF-kappaB activation by alternative splicing of MyD88. Curr Biol. 2002;12(6):467–71. doi: 10.1016/s0960-9822(02)00712-1 11909531

[12] Kugelberg E. Pattern recognition receptors: curbing gut inflammation. Nat Rev Immunol. 2014;14(9):583. doi: 10.1038/nri3735 25132096

[13] Mitchell G, Isberg RR. Innate Immunity to Intracellular Pathogens: Balancing Microbial Elimination and Inflammation. Cell Host Microbe. 2017;22(2):166–75. doi: 10.1016/j.chom.2017.07.005 28799902 PMC5562164

[14] Takeuchi O, Akira S. Pattern recognition receptors and inflammation. Cell. 2010;140(6):805–20. doi: 10.1016/j.cell.2010.01.022 20303872

[15] Sharma JM, Dohms JE, Metz AL. Comparative pathogenesis of serotype 1 and variant serotype 1 isolates of infectious bursal disease virus and their effect on humoral and cellular immune competence of specific-pathogen-free chickens. Avian Dis. 1989;33(1):112–24. doi: 10.2307/1591076 2539070

[16] Wang Q, Ou C, Wei X, Yu Y, Jiang J, Zhang Y, et al. CC chemokine ligand 19 might act as the main bursal T cell chemoattractant factor during IBDV infection. Poult Sci. 2019;98(2):688–94. doi: 10.3382/ps/pey435 30239915

[17] Bao K, Qi X, Li Y, Gong M, Wang X, Zhu P. Cryo-EM structures of infectious bursal disease viruses with different virulences provide insights into their assembly and invasion. Sci Bull (Beijing). 2022;67(6):646–54. doi: 10.1016/j.scib.2021.12.009 36546126

[18] Luque D, Rivas G, Alfonso C, Carrascosa JL, Rodríguez JF, Castón JR. Infectious bursal disease virus is an icosahedral polyploid dsRNA virus. Proc Natl Acad Sci U S A. 2009;106(7):2148–52. doi: 10.1073/pnas.0808498106 19164552 PMC2650107

[19] Mahgoub HA, Bailey M, Kaiser P. An overview of infectious bursal disease. Arch Virol. 2012;157(11):2047–57. doi: 10.1007/s00705-012-1377-9 22707044

[20] El-Aried TA, Mansour SMG, ElBakrey RM, N Ismail AE-S, Eid AAM. Infectious bursal disease virus: molecular epidemiologic perspectives and impact on vaccine efficacy against avian influenza and Newcastle disease viruses. Avian Dis. 2019;63(4):606–18. doi: 10.1637/aviandiseases-D-19-00086 31865675

[21] Winterfield RW, Hoerr FJ, Fadly AM. Vaccination against infectious bronchitis and the immunosuppressive effects of infectious bursal disease. Poult Sci. 1978;57(2):386–91. doi: 10.3382/ps.0570386 209433

[22] Hudson PJ, McKern NM, Power BE, Azad AA. Genomic structure of the large RNA segment of infectious bursal disease virus. Nucleic Acids Res. 1986;14(12):5001–12. doi: 10.1093/nar/14.12.5001 3014441 PMC311506

[23] Kibenge FS, McKenna PK, Dybing JK. Genome cloning and analysis of the large RNA segment (segment A) of a naturally avirulent serotype 2 infectious bursal disease virus. Virology. 1991;184(1):437–40. doi: 10.1016/0042-6822(91)90865-9 1651602

[24] Mundt E, Beyer J, Müller H. Identification of a novel viral protein in infectious bursal disease virus-infected cells. J Gen Virol. 1995;76 ( Pt 2)437–43. doi: 10.1099/0022-1317-76-2-437 7844565

[25] Fan L, Wang Y, Jiang N, Gao Y, Niu X, Zhang W, et al. Residues 318 and 323 in capsid protein are involved in immune circumvention of the atypical epizootic infection of infectious bursal disease virus. Front Microbiol. 2022;13:909252. doi: 10.3389/fmicb.2022.909252 35966653 PMC9372508

[26] Li Z, Qi X, Ren X, Cui L, Wang X, Zhu P. Molecular characteristics and evolutionary analysis of a very virulent infectious bursal disease virus. Sci China Life Sci. 2015;58(8):731–8. doi: 10.1007/s11427-015-4900-x 26245145

[27] Qi X, Gao X, Lu Z, Zhang L, Wang Y, Gao L, et al. A single mutation in the PBC loop of VP2 is involved in the in vitro replication of infectious bursal disease virus. Sci China Life Sci. 2016;59(7):717–23. doi: 10.1007/s11427-016-5054-1 27278372

[28] Qi X, Zhang L, Chen Y, Gao L, Wu G, Qin L, et al. Mutations of residues 249 and 256 in VP2 are involved in the replication and virulence of infectious Bursal disease virus. PLoS One. 2013;8(7):e70982. doi: 10.1371/journal.pone.0070982 23923037 PMC3724781

[29] Eldaghayes I, Rothwell L, Williams A, Withers D, Balu S, Davison F, et al. Infectious bursal disease virus: strains that differ in virulence differentially modulate the innate immune response to infection in the chicken bursa. Viral Immunol. 2006;19(1):83–91. doi: 10.1089/vim.2006.19.83 16553553

[30] Ruby T, Whittaker C, Withers DR, Chelbi-Alix MK, Morin V, Oudin A, et al. Transcriptional profiling reveals a possible role for the timing of the inflammatory response in determining susceptibility to a viral infection. J Virol. 2006;80(18):9207–16. doi: 10.1128/JVI.00929-06 16940532 PMC1563900

[31] Asfor AS, Nazki S, Reddy VRAP, Campbell E, Dulwich KL, Giotis ES, et al. Transcriptomic Analysis of Inbred Chicken Lines reveals infectious bursal disease severity is associated with greater bursal inflammation in vivo and more rapid induction of pro-inflammatory responses in primary bursal cells stimulated ex vivo. Viruses. 2021;13(5):933. doi: 10.3390/v13050933 34069965 PMC8157851

[32] Huang X, Liu W, Zhang J, Liu Z, Wang M, Wang L, et al. Very virulent infectious bursal disease virus-induced immune injury is involved in inflammation, apoptosis, and inflammatory cytokines imbalance in the bursa of fabricius. Dev Comp Immunol. 2021;114:103839. doi: 10.1016/j.dci.2020.103839 32898577

[33] Khatri M, Sharma JM. Infectious bursal disease virus infection induces macrophage activation via p38 MAPK and NF-kappaB pathways. Virus Res. 2006;118(1–2):70–7. doi: 10.1016/j.virusres.2005.11.015 16388870

[34] Li Y, Yang D, Jia Y, He L, Li J, Yu C, et al. Research Note: Anti-inflammatory effects and antiviral activities of baicalein and chlorogenic acid against infectious bursal disease virus in embryonic eggs. Poult Sci. 2021;100(4):100987. doi: 10.1016/j.psj.2021.01.010 33639350 PMC7921620

[35] Li YP, Handberg KJ, Juul-Madsen HR, Zhang MF, Jørgensen PH. Transcriptional profiles of chicken embryo cell cultures following infection with infectious bursal disease virus. Arch Virol. 2007;152(3):463–78. doi: 10.1007/s00705-006-0878-9 17143781

[36] Xu Z-Y, Yu Y, Liu Y, Ou C-B, Zhang Y-H, Liu T-Y, et al. Differential expression of pro-inflammatory and anti-inflammatory genes of layer chicken bursa after experimental infection with infectious bursal disease virus. Poult Sci. 2019;98(11):5307–14. doi: 10.3382/ps/pez312 31222288

[37] Zhang S, Zheng S. Host Combats IBDV Infection at Both Protein and RNA Levels. Viruses. 2022;14(10):2309. doi: 10.3390/v14102309 36298864 PMC9607458

[38] Geissmann F, Manz MG, Jung S, Sieweke MH, Merad M, Ley K. Development of monocytes, macrophages, and dendritic cells. Science. 2010;327(5966):656–61. doi: 10.1126/science.1178331 20133564 PMC2887389

[39] Li Y, Yu P, Kessler AL, Shu J, Liu X, Liang Z, et al. Hepatitis E virus infection activates NOD-like receptor family pyrin domain-containing 3 inflammasome antagonizing interferon response but therapeutically targetable. Hepatology. 2022;75(1):196–212. doi: 10.1002/hep.32114 34392558 PMC9299901

[40] Chen L-F, Greene WC. Shaping the nuclear action of NF-kappaB. Nat Rev Mol Cell Biol. 2004;5(5):392–401. doi: 10.1038/nrm1368 15122352

[41] Lawrence T. The nuclear factor NF-kappaB pathway in inflammation. Cold Spring Harb Perspect Biol. 2009;1(6):a001651. doi: 10.1101/cshperspect.a001651 20457564 PMC2882124

[42] Chen ZJ. Ubiquitin signalling in the NF-kappaB pathway. Nat Cell Biol. 2005;7(8):758–65. doi: 10.1038/ncb0805-758 16056267 PMC1551980

[43] Hayden MS, Ghosh S. Shared principles in NF-kappaB signaling. Cell. 2008;132(3):344–62. doi: 10.1016/j.cell.2008.01.020 18267068

[44] Scheidereit C. IkappaB kinase complexes: gateways to NF-kappaB activation and transcription. Oncogene. 2006;25(51):6685–705. doi: 10.1038/sj.onc.1209934 17072322

[45] Warner N, Núñez G. MyD88: a critical adaptor protein in innate immunity signal transduction. J Immunol. 2013;190(1):3–4. doi: 10.4049/jimmunol.1203103 23264668

[46] Deng L, Wang C, Spencer E, Yang L, Braun A, You J, et al. Activation of the IkappaB kinase complex by TRAF6 requires a dimeric ubiquitin-conjugating enzyme complex and a unique polyubiquitin chain. Cell. 2000;103(2):351–61. doi: 10.1016/s0092-8674(00)00126-4 11057907

[47] Nagpal K, Plantinga TS, Sirois CM, Monks BG, Latz E, Netea MG, et al. Natural loss-of-function mutation of myeloid differentiation protein 88 disrupts its ability to form Myddosomes. J Biol Chem. 2011;286(13):11875–82. doi: 10.1074/jbc.M110.199653 21325272 PMC3064238

[48] Lin S-C, Lo Y-C, Wu H. Helical assembly in the MyD88-IRAK4-IRAK2 complex in TLR/IL-1R signalling. Nature. 2010;465(7300):885–90. doi: 10.1038/nature09121 20485341 PMC2888693

[49] Motshwene PG, Moncrieffe MC, Grossmann JG, Kao C, Ayaluru M, Sandercock AM, et al. An oligomeric signaling platform formed by the Toll-like receptor signal transducers MyD88 and IRAK-4. J Biol Chem. 2009;284(37):25404–11. doi: 10.1074/jbc.M109.022392 19592493 PMC2757241

[50] Qi X, Gao H, Gao Y, Qin L, Wang Y, Gao L, et al. Naturally occurring mutations at residues 253 and 284 in VP2 contribute to the cell tropism and virulence of very virulent infectious bursal disease virus. Antiviral Res. 2009;84(3):225–33. doi: 10.1016/j.antiviral.2009.09.006 19766142

[51] Niu X, Han J, Huang M, Wang G, Zhang Y, Zhang W, et al. Infectious bursal disease virus VP5 triggers host shutoff in a transcription-dependent manner. mBio. 2024;15(3):e0343323. doi: 10.1128/mbio.03433-23 38289089 PMC10936426

[52] Liu A, Li H, Qi X, Wang Q, Yang B, Wu T, et al. Macrophage migration inhibitory factor triggers inflammatory responses during very virulent infectious bursal disease virus infection. Front Microbiol. 2019;10:2225. doi: 10.3389/fmicb.2019.02225 31632367 PMC6779731

[53] Jaunin F, Burns K, Tschopp J, Martin TE, Fakan S. Ultrastructural distribution of the death-domain-containing MyD88 protein in HeLa cells. Exp Cell Res. 1998;243(1):67–75. doi: 10.1006/excr.1998.4131 9716450

[54] O’Connell CM, Ionova IA, Quayle AJ, Visintin A, Ingalls RR. Localization of TLR2 and MyD88 to Chlamydia trachomatis inclusions. Evidence for signaling by intracellular TLR2 during infection with an obligate intracellular pathogen. J Biol Chem. 2006;281(3):1652–9. doi: 10.1074/jbc.M510182200 16293622

[55] Loiarro M, Gallo G, Fantò N, De Santis R, Carminati P, Ruggiero V, et al. Identification of critical residues of the MyD88 death domain involved in the recruitment of downstream kinases. J Biol Chem. 2009;284(41):28093–103. doi: 10.1074/jbc.M109.004465 19679662 PMC2788860

[56] Rebl A, Rebl H, Köbis JM, Goldammer T, Seyfert H-M. ST2 from rainbow trout quenches TLR signalling, localises at the nuclear membrane and allows the nuclear translocation of MYD88. Dev Comp Immunol. 2017;67:139–52. doi: 10.1016/j.dci.2016.10.009 27776995

[57] Li L, Yang W, Ma X, Wu J, Qin X, Cao X, et al. Peste des petits ruminants virus N protein is a critical proinflammation factor that promotes MyD88 and NLRP3 complex assembly. J Virol. 2022;96(10):e0030922. doi: 10.1128/jvi.00309-22 35502911 PMC9131857

[58] Ahn M, Chen VC-W, Rozario P, Ng WL, Kong PS, Sia WR, et al. Bat ASC2 suppresses inflammasomes and ameliorates inflammatory diseases. Cell. 2023;186(10):2144-2159.e22. doi: 10.1016/j.cell.2023.03.036 37172565

[59] Jennings E, Esposito D, Rittinger K, Thurston TLM. Structure-function analyses of the bacterial zinc metalloprotease effector protein GtgA uncover key residues required for deactivating NF-κB. J Biol Chem. 2018;293(39):15316–29. doi: 10.1074/jbc.RA118.004255 30049795 PMC6166728

[60] Keestra AM, de Zoete MR, Bouwman LI, Vaezirad MM, van Putten JPM. Unique features of chicken Toll-like receptors. Dev Comp Immunol. 2013;41(3):316–23. doi: 10.1016/j.dci.2013.04.009 23628643

[61] Qi X, Gao L, Qin L, Deng X, Wu G, Zhang L. Genomic sequencing and characterization of a very virulent strain of infectious bursal disease virus isolated in China. Agricultural science & technology. 2011;12(12):1946–9. doi: 10.16175/j.cnki.1009-4229.2011.12.004

[62] Wang Y, Jiang N, Fan L, Gao L, Li K, Gao Y, et al. Development of a Viral-Like Particle Candidate Vaccine Against Novel Variant Infectious Bursal Disease Virus. Vaccines (Basel). 2021;9(2):142. doi: 10.3390/vaccines9020142 33579020 PMC7916800

