# Supplementary figures and images for: Enhancing MyD88 oligomerization is one important mechanism by which IBDV VP2 induces inflammatory response
Source: PLoS Pathog. 2025 Mar 11;21(3):e1012985. doi: 10.1371/journal.ppat.1012985 (PMC11957393; doi:10.1371/journal.ppat.1012985)

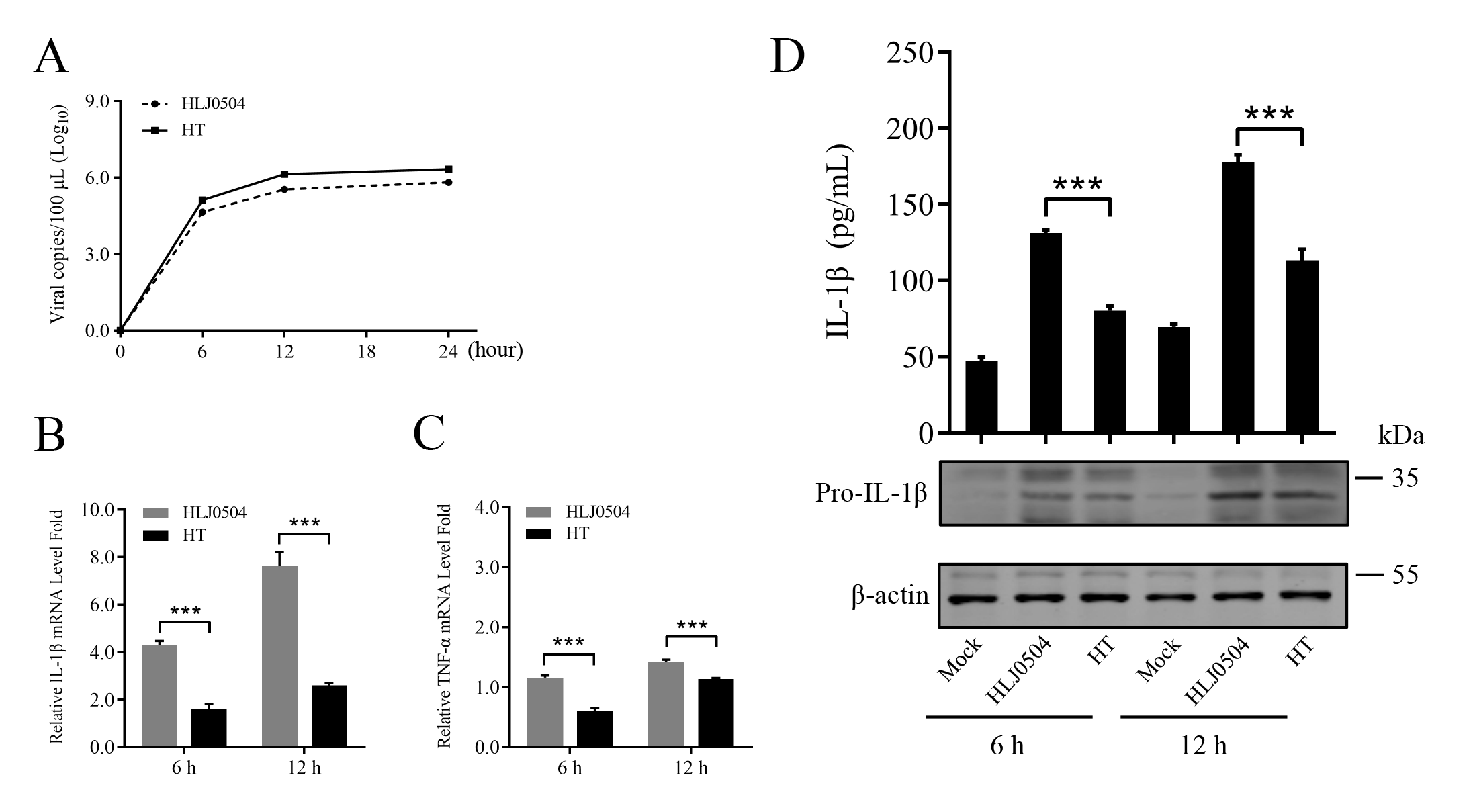

Supplement: S1 Fig — (A) The growth dynamics of the HLJ0504 and HT strain of IBDV in DT40 cells were analysed using RT-qPCR. (B-D) Effect of HLJ0504 or HT strain on the expression of IL-1β, TNF-α, and production of IL-1β. DT40 cells were infected with HLJ0504 or HT strain (1×10^10^ copies/1×106 cells) for 6 and 12 hpi, and levels of IL-1β (B) and TNF-α (C) mRNA were assessed by RT-qPCR. The pro-IL-1β in the cell lysates were analysed by immunoblot analysis, and IL-1β in the cell supernatants were measured by ELISA (D). All data are representative of at least three independent experiments. Graphs show mean ± SD, n=3, ***, P<0.001. (TIF) [file ppat.1012985.s001.tif]
